# The role of somatosensation in automatic visuo-motor control: a comparison of congenital and acquired sensory loss

**DOI:** 10.1007/s00221-021-06110-y

**Published:** 2021-04-28

**Authors:** R. Chris Miall, Daria Afanasyeva, Jonathan D. Cole, Peggy Mason

**Affiliations:** 1 School of Psychology, University of Birmingham, Birmingham, B15 2TT UK; 2 Centre of Postgraduate Research and Education, Bournemouth University, Bournemouth, UK; 3 Department of Neurobiology, The University of Chicago, Chicago, IL USA

**Keywords:** Somatosensation, Automaticity, Human movement, Proprioception, Drawing, Writing, Mirror-tracing

## Abstract

Studies of chronically deafferented participants have illuminated how regaining some motor control after adult-onset loss of proprioceptive and touch input depends heavily on cognitive control. In this study we contrasted the performance of one such man, IW, with KS, a woman born without any somatosensory fibres. We postulated that her life-long absence of proprioception and touch might have allowed her to automate some simple visually-guided actions, something IW appears unable to achieve. We tested these two, and two age-matched control groups, on writing and drawing tasks performed with and without an audio-verbal echoing task that added a cognitive demand. In common with other studies of skilled action, the dual task was shown to affect visuo-motor performance in controls, with less well-controlled drawing and writing, evident as increases in path speed and reduction in curvature and trial duration. We found little evidence that IW was able to automate even the simplest drawing tasks and no evidence for automaticity in his writing. In contrast, KS showed a selective increase in speed of signature writing under the dual-task conditions, suggesting some ability to automate her most familiar writing. We also tested tracing of templates under mirror-reversed conditions, a task that imposes a powerful cognitive planning challenge. Both IW and KS showed evidence of a visuo-motor planning conflict, as did the controls, for shapes with sharp corners. Overall, IW was much faster than his controls to complete tracing shapes, consistent with an absence of visuo-proprioceptive conflict, whereas KS was slower than her controls, especially as the corners became sharper. She dramatically improved after a short period of practice while IW did not. We conclude that KS, who developed from birth without proprioception, may have some visually derived control of movement not under cognitive control, something not seen in IW. This allowed her to automate some writing and drawing actions, but impaired her initial attempts at mirror-tracing. In contrast, IW, who lost somatosensation as an adult, cannot automate these visually guided actions.

**Supplementary Information:**

The online version contains supplementary material available at 10.1007/s00221-021-06110-y.

## Introduction

Comparison of congenital and acquired neurological conditions can provide profound insight into the development of neurological function. In this paper, we contrast the limits of fine motor control in two participants with very rare yet related neurological conditions: a man with acquired, adult-onset degeneration of large sensory fibres, resulting in the loss of proprioception and touch, and a woman with the complete absence of large and small sensory fibres from birth. The comparison of these two rare individuals provides us with the unique opportunity to uncover, for the first time, the role of somatosensory information in sensorimotor function in the developing body and how other sensory modalities and cognition can develop, maintain and adapt visuo-motor function over decades.

The acquired loss of touch and movement/position sense from the body is thought to involve an auto-immune response to an infection, resulting in the degeneration of large myelinated sensory fibres (Cole 1995). A small number of individuals with this rare condition have been extensively tested in laboratories around the world, providing valuable observations into the control of posture and movement and other sensorimotor and cognitive functions, in the absence of normal large fibre input. IW, a man now in his sixties who developed large-fibre sensory loss at age 19 (Cole and Sedgwick 1992) and who has lived without the senses of touch or proprioception from below the lower face or neck, is compared to KS, a woman tested at age 40, who was born without any somatosensory fibres. KS’s condition was documented by both biopsies and electrodiagnostic tests before the age of three. Her lack of both large and small sensory fibres in lumbar, cervical and trigeminal dermatomes was confirmed within the past 5 years by neurological exam and nerve conduction studies (Mason et al., manuscript in preparation). She is the only known living person with a total congenital somatosensory defect; a full description of her condition is in preparation.

Adult-onset deafferentation has left IW highly reliant on vision, cognition and attention to control his actions (Cole 1995, 2016; Ingram et al. 2000). His movements have thus little or no automaticity and without attention he loses accuracy and adaptability. A similar case, GL, showed impaired writing skill, with a lack of automaticity and dependence on vision (Hepp-Reymond et al. 2009; Danna and Velay 2017). In contrast, our observations of KS, who has lacked somatosensation since birth, hinted that she appears to perform some movements with a degree of automaticity and without the constraint of conscious visual supervision. Given her development, we postulated that she may be able to use sub-conscious (potentially sub-cortical) visual inputs, such as those that target the midbrain, to control her movement (Reynolds and Day 2012).

Our aim, therefore, was to illuminate the extent to which these two deafferented people, IW and KS, with acquired or congenital deafferentation, respectively, can act automatically. We chose handwriting and drawing as actions that are normally under visual guidance, but also show some degree of automaticity. Our test of automaticity is based on the changes in performance seen in control participants when writing or drawing during concurrent performance of a secondary, cognitively challenging, task: verbal echoing of an audiobook. At first glance, adding a secondary task may be expected to reduce the processing capacity available for the primary task, and thus lead to degraded primary task performance. While this is true for many cognitive tasks, and for novel motor tasks (Ingram et al. 2000; Galea et al. 2010), it is not always seen with or during expert motor tasks. For well-learned actions, including those involved in playing sports, allocating attention to a secondary task can actually improve performance (Beilock et al. 2002, 2004). Hence, we interpreted degradation in performance under dual-task conditions as a marker of low automaticity, and no change or improvement to performance as signs of automaticity.

We chose visuo-motor tasks that differed in complexity and expected automaticity. For example, writing single letters may be automated in accomplished writers (Tucha and Lange 2005), as may some simple and regularly used words; more complex words and phrases require more top–down control (Wing 2000; Tucha et al. 2006; MacMahon and Charness 2014). Signatures have a highly stereotyped trajectory, and are written automatically; they can be recognisably reproduced, for example, at very different scales requiring either distal (finger) or proximal (whole arm) muscles (Wing 2000). Shapes of progressive complexity from circles to five-pointed stars, marked by an increase in acute angles, require progressively greater top–down control. The effect of a dual task on this range of visuo-motor tasks was then used to probe automaticity.

In addition, we looked at the effect of visually reversed feedback on tracing shapes in a mirror. In controls, this task imposes conflicts between vision, intended action and proprioceptive feedback. The mirror-reversal causes a planning conflict between vision of the template and the necessary direction of pen movement, most noticeable when sharp changes in direction are required (e.g. vision might dictate a movement southeast but the hand must move northeast). There is also a conflict between visual and proprioceptive feedback, as the movements made evoke visual feedback that conflicts with expectations. However, over repeated attempts, participants improve their performance through a learned process.

Lajoie et al. (1992) found that GL, an individual who, like IW, lost touch and proprioception as an adult, was able to quickly trace a Star of David on her initial try. They concluded this was because GL had no conflict between vision and (her absent) proprioceptive reafference. Subsequently, Miall and Cole (2007) found that IW could mirror-trace smooth shapes faster than controls, but like the controls, he was impeded at corners. They concluded that he experienced a planning conflict between vision and his feedforward motor programme. Notably, IW suffered a motor planning conflict but GL did not; reflecting their two different approaches to the task. We expected that, similar to IW and controls, KS would experience a forward planning conflict but, as was true of IW, she would not be affected by a conflict between (absent) proprioception and vision. We further hypothesised that KS might perform more poorly than IW at mirror-tracing because her actions would be more automatically driven by visual inputs. Thus, she might experience both a forward, cognitive, planning conflict, but also a (potentially subconscious) conflict in processing reversed visual feedback. We will return to this issue in the Discussion. To address this, we asked IW and KS to mirror-trace shapes that ranged from circles to polygons with multiple acute angles.

Responding to KS’s enjoyment of the mirror-tracing task, we added an experiment to determine how IW’s and KS’s mirror-tracing performance changed after practice. Short-term visuo-motor adaptation may depend on subconscious processes that lead to learned actions that are fast and automatic or, in contrast, may result from the adoption of slow cognitive strategies (Taylor et al. 2010; Taylor and Ivry 2014). Our prediction for IW was the latter, after practice, whereas we hypothesised that KS would show signs of fast, automatic actions.

## Materials and methods

### Participants

Two participants with profound somatosensory loss were tested. We refer to these two individuals as ‘deafferented’ or test participants, to be compared with control groups of age-matched, neurologically normal participants. First, IW, a 66-year-old male with an acquired large sensory fibre deafferentation (Cole 1995), has no sense of light touch nor movement/position sense from below a level at the collar line anteriorly and extending to the top of the head posteriorly (C3 spinal level). Temperature and pain perception are intact, as is motor nerve function, verified from nerve conduction studies and EMG (Cole and Katifi 1991; Cole and Sedgwick 1992). The other test participant, KS, is a 40-year-old female with a congenital loss of all somatosensory inputs over her whole body. She has no sensory fibres, either myelinated or unmyelinated, as determined by multiple modes of testing—nerve conduction, biopsy, and evoked potentials as well as by clinical/neurological testing (Mason, Axelrod, Rezania, and Reder, unpublished observations).

Because of the age difference of the two deafferented participants, two separate groups of control participants were recruited. IW was matched with seven controls with a mean age of 67.4 years (SD = 3.63, three males, four females) and KS with seven controls of mean age 38.4 years (SD = 3.71, four males, three females). The experiments reported here were conducted at the same time as others, using the same control groups (Miall et al. 2021). Written informed consent was obtained for each participant prior to the study which was approved by the University of Birmingham ethics board, and performed in accordance with the Declaration of Helsinki.

All participants, test and control, were fluent English speakers and had normal or corrected-to-normal vision and hearing. To confirm that the control participants had normal hand function, they completed the 9-hole peg test and their average performance times were cross-referenced to standardised values (Oxford Grice et al. 2003). It should be noted that the 9-hole peg hole test requires distal fine motor skills; the writing and drawing tasks included in the present experiments can be accomplished with more proximal muscles as long as the drawing stylus can be held in a steady grasp. Both KS and IW have deficits in everyday use of their hands (Miall et al. 2021) and neither of them handwrite frequently. IW uses a “cross-thumb” grasp for greater stability when holding a pen (Miall et al. 2019), while KS uses a whole-hand power grip.

All participants also completed the 10-item Edinburgh Handedness Inventory (Oldfield 1971). The two deafferented participants are strongly left-handed (Edinburgh handedness inventory scores: KS: − 80 AND IW: − 100). The older control group included one left-hander with a score of − 95; all other control participants were right-handed with scores of + 100. To inform the comparison between controls and the test participants who wrote and draw infrequently, all the control participants performed all tasks with both dominant and non-dominant hands. A standard order of left and then right hand was used, regardless of handedness. The deafferented participants used only their preferred left hand except where noted.

### Experiments: writing, drawing and mirror-tracing

We used a Summagraphics Summasketch digitising board, with a writing area of 30 × 30 cm, which was placed flat on a table in front of the participant. The stylus did not leave any visible mark on the writing surface and only reported when the spring-loaded tip was in contact with the surface. Data collection from the digitising board was read and stored on a computer for subsequent analysis using a bespoke Matlab script (Mathworks, Natick, MA, USA).

The deafferented participants sat in front of the board in their wheelchairs while control participants used a standard office chair. All participants performed three separate tasks, of which Experiments 1 and 2 were performed both with and without a dual task, to add an additional cognitive load. During the dual-task participants listened to, and echoed out loud, an audiobook (HG Wells’ “War of the Worlds” or Anna Sewell’s “Black Beauty”) that was played over headphones. Tasks were completed first without and then with the dual task.

#### Experiment 1: drawing task

In each 30 s trial, participants continuously, without interruption, drew an instructed shape from memory (a circle, square or a five-pointed star, chosen as simple shapes with increasing complexity because of the acute angles). They were asked to repeat each shape as accurately as possible, both in shape and size for as many cycles as possible within the 30 s trial period. They saw their hand on the digitising tablet, but the stylus left no mark. They drew each shape with and without the dual-task. To increase the sample, we tested IW and KS on this task (with and without the dual task) on both Day 1 and Day 2, and then on Day 3 they drew another three shapes (jelly bean, triangle and diamond), with similarly increasing complexity in the number and sharpness of the corners to the original shapes. For comparison with the control groups, we averaged the deafferented participants’ performance measures from the corresponding circle/jellybean, square/triangle and star/diamond tasks across the three sessions.

#### Experiment 2: signature and writing task

 Control participants performed three short writing tasks: writing first their signature, next their name (in either cursive or print as they preferred), and finally a two-word phrase such as “Friday morning.” They executed all three forms of writing in both the normal (left to right) and reversed direction (right to left), with both dominant and non-dominant hand, and with and without the added cognitive load. Including performances with the non-dominant hand, as well as dominant hand, for the control participants was designed to inform the comparison between the control and deafferented participants, given the relatively infrequent use of handwriting by the latter. The order of the 24 writing tasks was fixed across control participants.

The two deafferented participants also wrote their signature and their name (IW using print, KS using a cursive script by choice). They each wrote the phrase “Monday morning” and also two additional 2-word phrases chosen to be similar in letter count and identity to their own names. They performed each of these tasks with and without the dual task. IW and KS performed all tasks with their dominant hand only.

#### Experiment 3: mirror-reversed tracing

**Fig. 1:**
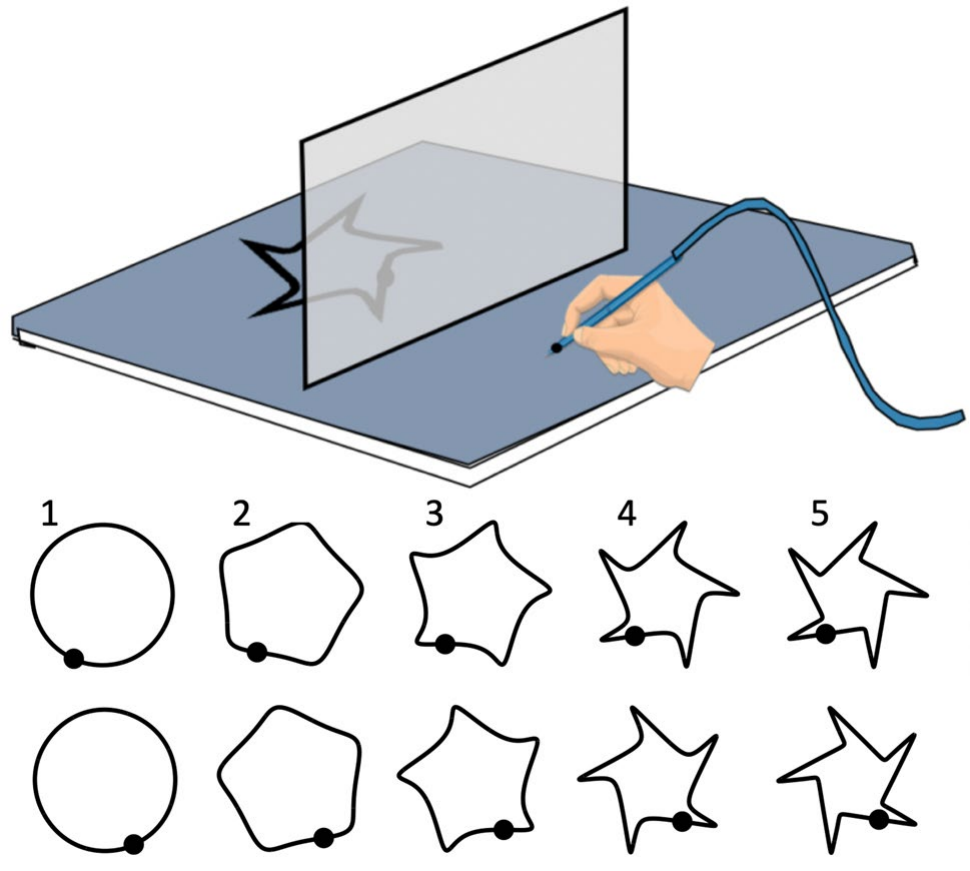
The set-up used for the mirror-tracing task (Experiment 3). For each trial one of the templates was placed behind the semi-silvered mirror and participants traced it on the board in front, starting at the point indicated by the filled symbol. Templates in the top row were traced in a clockwise direction and the mirror-symmetric images in the bottom row were traced anti-clockwise. For the writing and drawing tasks, the vertical mirror was removed, and the whole of the drawing surface was available

Ten template shapes (five left–right reversed pairs) of varying complexity were printed on A4 paper. Templates were placed behind a vertically oriented semi-silvered front-surfaced mirror on the digitising board (Fig. 1). Each template had a clearly marked starting point such that the virtual image in the mirror aligned with a fixed position in front of the mirror on the drawing surface. At the start of each trial the participant placed the stylus on a start mark on the drawing board as the template was moved into position, such that the stylus was aligned in the mirror with the start point of the template. When ready, they traced the shape as quickly and accurately as possible, aiming to complete one cycle of the shape within the 2-min trial. The control participants traced shapes from 1 to 5, one-by-one; the sequence was then repeated, with the second set of templates, while they performed the dual echoing task; they then repeated this sequence using their non-dominant hand.

The two deafferented participants traced the same sets of 5 shapes, using only their dominant hand, and repeated this on two successive days. We averaged performance over these two days before comparison with the controls. After completing the mirror-tracing on Day 1, they also directly traced on top of the shapes, without the vertical mirror, to allow us to assess their speed and accuracy during normal tracing.

On Day 3, encouraged by their interest in the mirror-tracing task, we gave KS and IW a total of about 10 min practice in tracing a variety of different simple shapes (squares, figure-of-eights in both orientations, and a word printed in large block letters). Following training, we tested their performance on the original set of five shapes (Fig. 1), with dominant and then non-dominant hands. Comparisons of pre- and post-training data were made only for the two deafferented participants.

### Data analysis

**Fig. 2:**
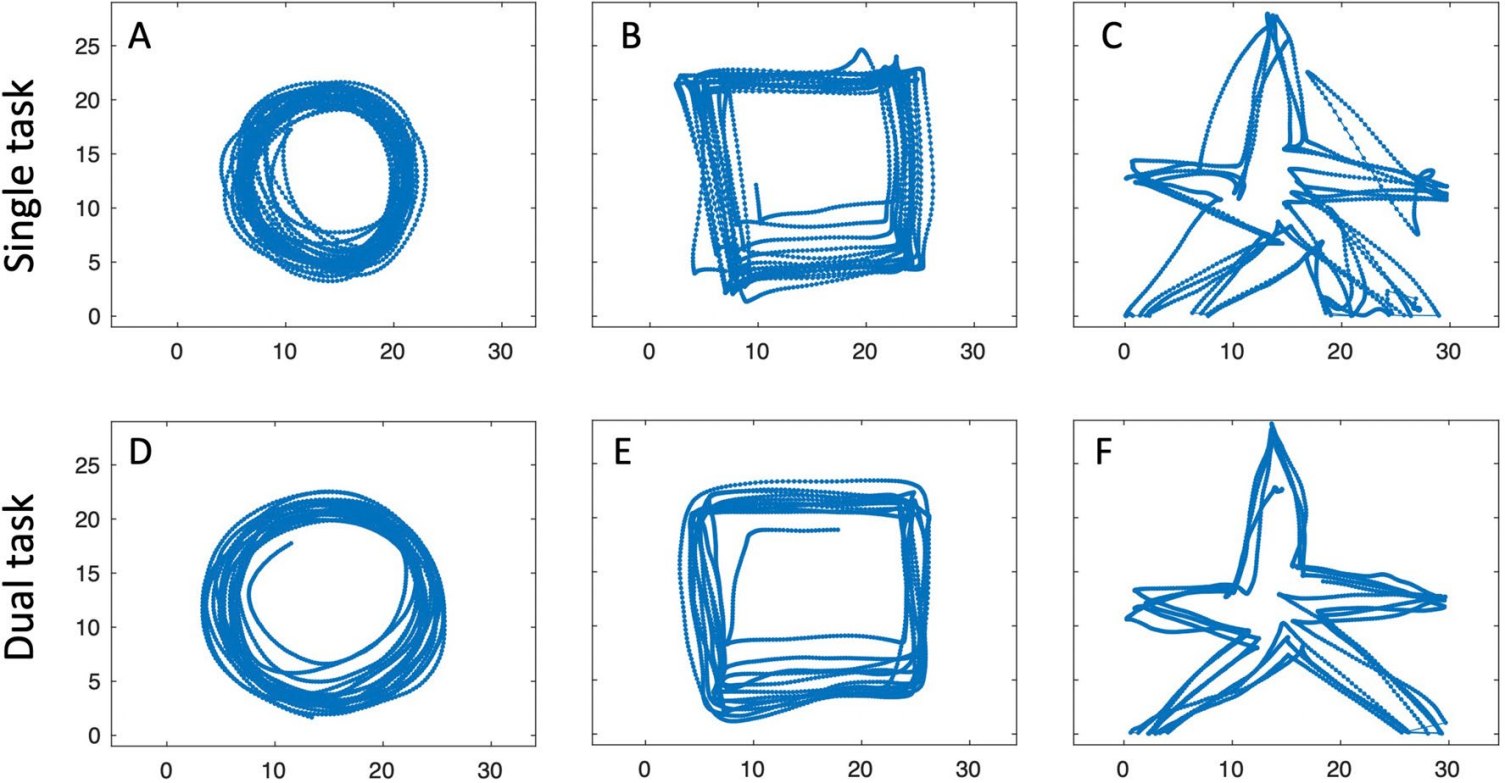
IW drawing. Top row: single task condition. Bottom row: somewhat larger drawings under the dual-task condition. The changes in size tended to be systematic, gradually increasing as the trial continued

The digitising board sampled the stylus position in centimetres, at a frequency of 120 samples per second and with a resolution of ~ 200 samples/cm (500/inch). The stylus location was only recorded when the stylus tip was in direct contact with the drawing surface. Both IW and KS (and all controls) were able to keep the pen in contact with the surface; on a few occasions IW and KS slightly overshot the active drawing area and the pen position was reported as a constant (see Fig. 2c, f for examples). For time-based measures, all data were analysed in the native resolution of 120 samples/s after low pass filtering with 4-pole Butterworth filter, cut-off 20 Hz.

First, for every trial, the raw time-series of X and Y data-points was visually inspected to determine movement onset and offset (trial duration). This was determined by either the moments when the pen abruptly started or stopped moving, or the pen was lifted from the writing board. We report trial duration for Experiments 2 and 3; in Experiment 1 trial duration was fixed at 30 s.

Two measures of the stylus motion were then computed for all tasks: (i) pathlength of the drawn shape, i.e. the total length of the drawn line when in contact with the surface (in cm); (ii) mean path speed across the whole trial (pathlength/duration, cm/s). In addition, for Experiments 1 and 2 we measured mean curvature (cm^−1^, the reciprocal of the radius). This provides an estimate of jerkiness of movement, since higher curvature values reflect less fluid cursive writing and sharper corners. The radius of arcs fitted to each triplet of position samples (three successive positions) were found, and their reciprocals averaged.

In Experiment 1 we calculated the mean pathlength per cycle of the repeated drawing after segmentation with an automatic algorithm that was visually checked to ensure consistency across the cycles. We also calculated a goodness of fit (GoF); this ranges from 0 to 1, analogous to the r-squared value of a correlation and quantified the spatial match of each drawn shape to the previous iteration of the same shape. Goodness of fit was estimated with the Procrustes function in Matlab, allowing spatial relocation and rotation of each shape, but without scaling or reflexion of the shape.

In Experiment 3, we calculated the pathlength (cm) and mean error (cm) estimated as the mean distance between the stylus location and the nearest point on the template after realignment with the Procrustes transform to minimise global error.

For the spatial measures (curvature, goodness of fit, and error), each time-series was spatially resampled prior to the analysis, to give 1000 equally-spaced samples along the original trajectory.

### Statistics

Our strategy was to first compare the two control groups, for each task, using mixed ANOVA tests with factors (control group: younger/older) × (hand: dominant/non-dominant) × cognitive load (single/dual task) × word or shape (with 2/3/5 levels, depending on the task), for each computed measurement (see Supplementary Materials for the results). This was to test for any significant differences between the two control groups that were separately aged-matched to the deafferented participants. We also explored whether there were systematic differences in performance between the two hands, and whether the performance was affected by an added cognitive load. Where necessary (significant Mauchly’s test for sphericity) we report the Greenhouse–Geisser corrected degrees of freedom and probability.

Following control group analyses, we compared the two deafferented participants to their respective control group using *Q*´ tests (Michael 2007; Renault et al. 2018; Bartolo et al. 2018; Miall et al. 2021). Many tests exist to compare single cases to a group; we chose the Q’ test to allow comparison of IW and KS to their separate control groups in a 2 × 3 factorial design (Experiments 1, 2) or 2 × 5 factorial design (Experiment 3). The Q’ tests can compare the case and control group across any number of tests, adjusting the *z*-score to reflect the differences from the mean across all conditions and participants. Thus, in Experiments 1 and 2 we calculated the difference in each participant’s performance measure in the single versus dual-task conditions. The test participants’ (IW and KS) difference scores were then transformed into *Z*-scores, based on the mean and sample standard deviation of the corresponding control group differences; providing a case–control comparison for each condition tested, reported here as *q*´ (lower case). Unlike other case–control tests, the *Q*´ tests also allow analysis of the interaction between factors. The factorial *Q*´-test reveals whether the pattern of performance differences across the main factor of each experiment was significantly different for the case (KS or IW) from the pattern for the controls. In other words, this allowed a test of main factor (word/shape) on the cognitive load imposed by the dual task for the test participants relative to their respective controls. The *Q*´ and *q*´ tests did not, however, allow us to statistically compare the two deafferented participants with each other. For figures, we present error bars as the 95% confidence limits (1.96 × SEM, based on the sample SD).

## Results

### Experiment 1—repeated drawing

This experiment involved repeated drawing of three shapes with vision of the hand, but without a visible trace on the tablet, with and without the verbal echoing dual task. We hypothesised that the increase in complexity of the drawn shapes, from circle to square to star, would be reflected in reduced speed and consistency (the goodness of fit from one cycle to the next), and that an added cognitive load would preferentially compromise the more cognitively demanding and less automated shapes; whereas, the drawing of circles, which were likely to be most automatically controlled, would be relatively spared for all groups.

**Fig. 3:**
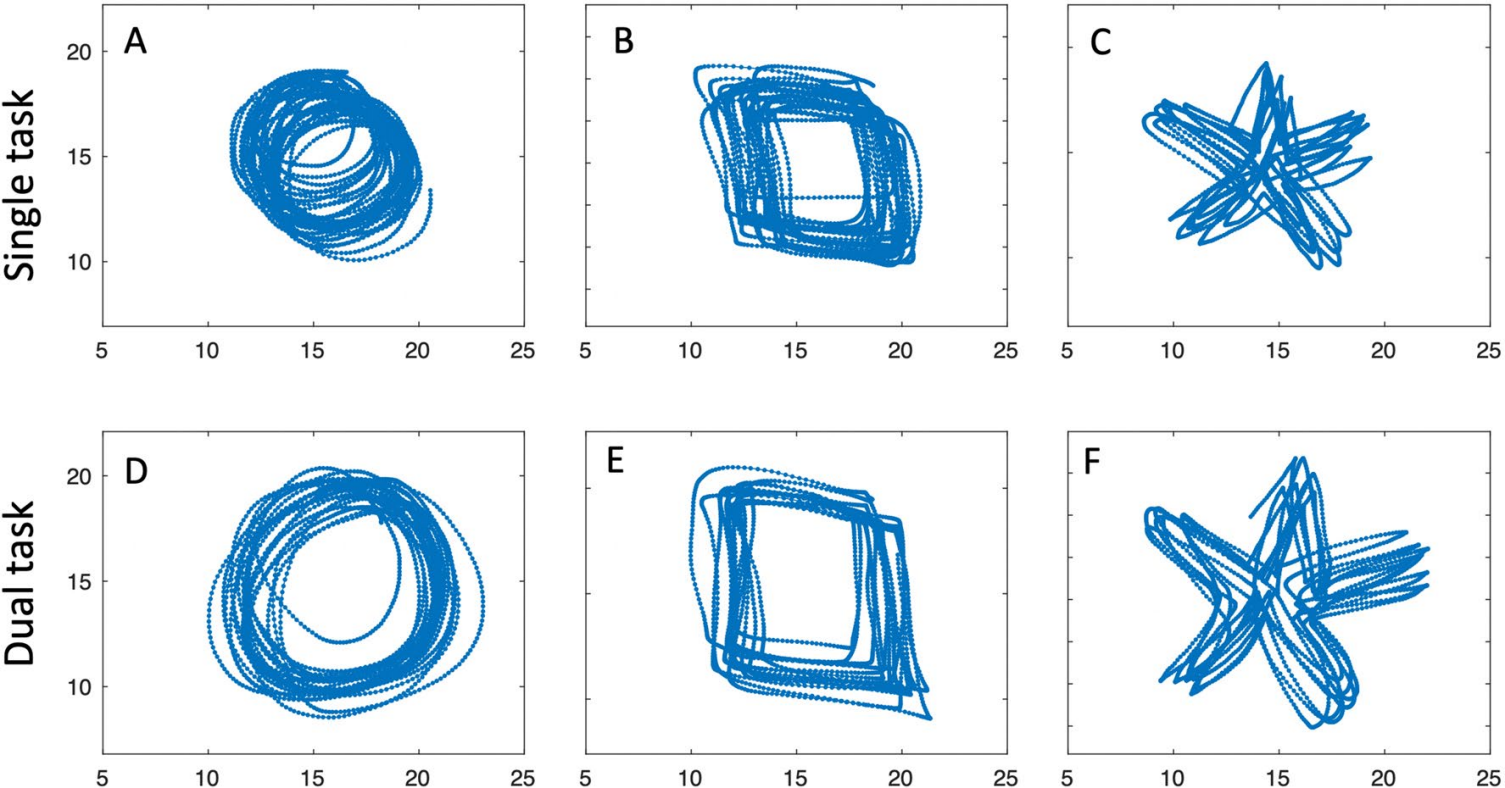
KS drawing. Same conventions as Fig. 2; note the scale (KS’s drawings were about 60% smaller than IW’s). As with IW, size tended to increase across the duration of each trial, and there was sometimes a drift in location (e.g. in panels **a**, **b** and **e**)

In Figs. 2 and 3 we show examples of IW and KS drawing the three shapes, with and without the dual task, tested only with their dominant hands.

#### Control participants

**Fig. 4:**
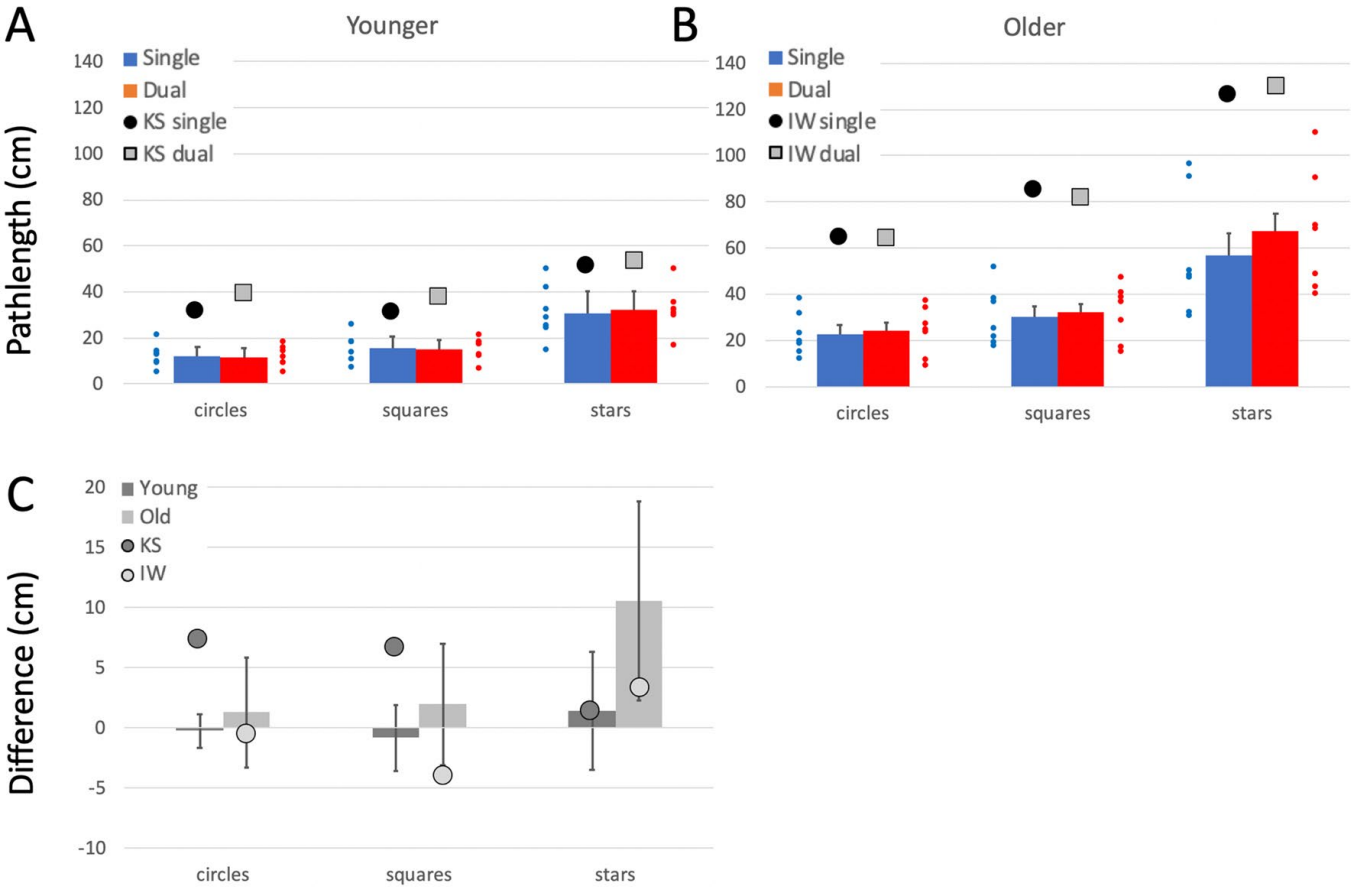
**a**/**b** Pathlength per cycle (cm), on the free shape-drawing task. Blue bars are the control group means (dominant hand only, error bars: 95% confidence limits for the control groups; *n* = 7) in single task conditions; the small blue dots are individual participants. Red bars and small red dots are under the dual task. The large black dots are the mean data from IW **a** and KS **b** in single task conditions; the large grey squares are their performance under dual-task conditions. **c** Difference in pathlength from single to dual-task conditions. The younger controls and KS are in dark grey; the older controls and IW are in light grey

**Fig. 5:**
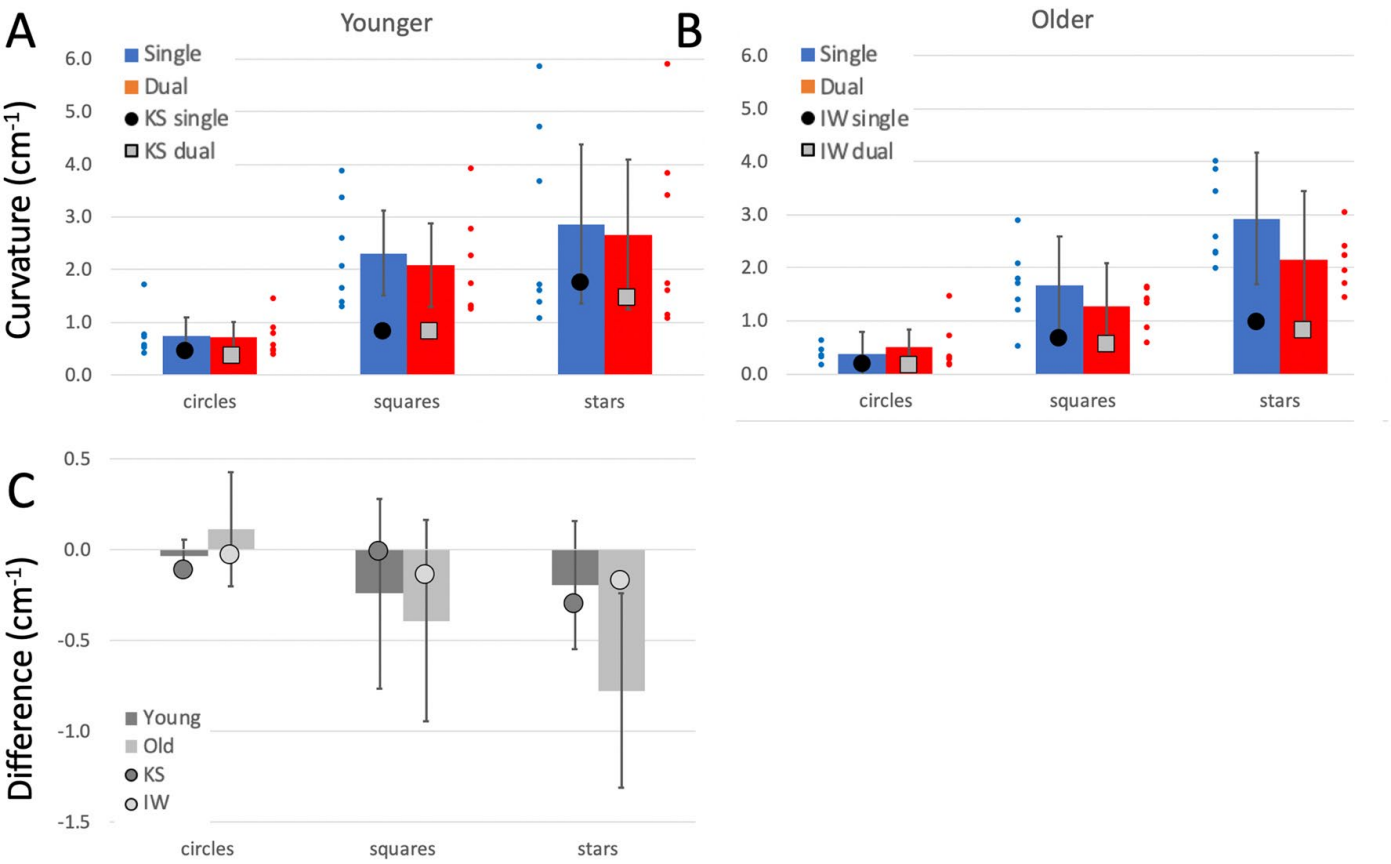
**a, b** Curvature in the shape-drawing tasks. Units in cm^−1^. **c** Difference in curvature from single to dual-task conditions. Format is as in Fig. 4

**Fig. 6:**
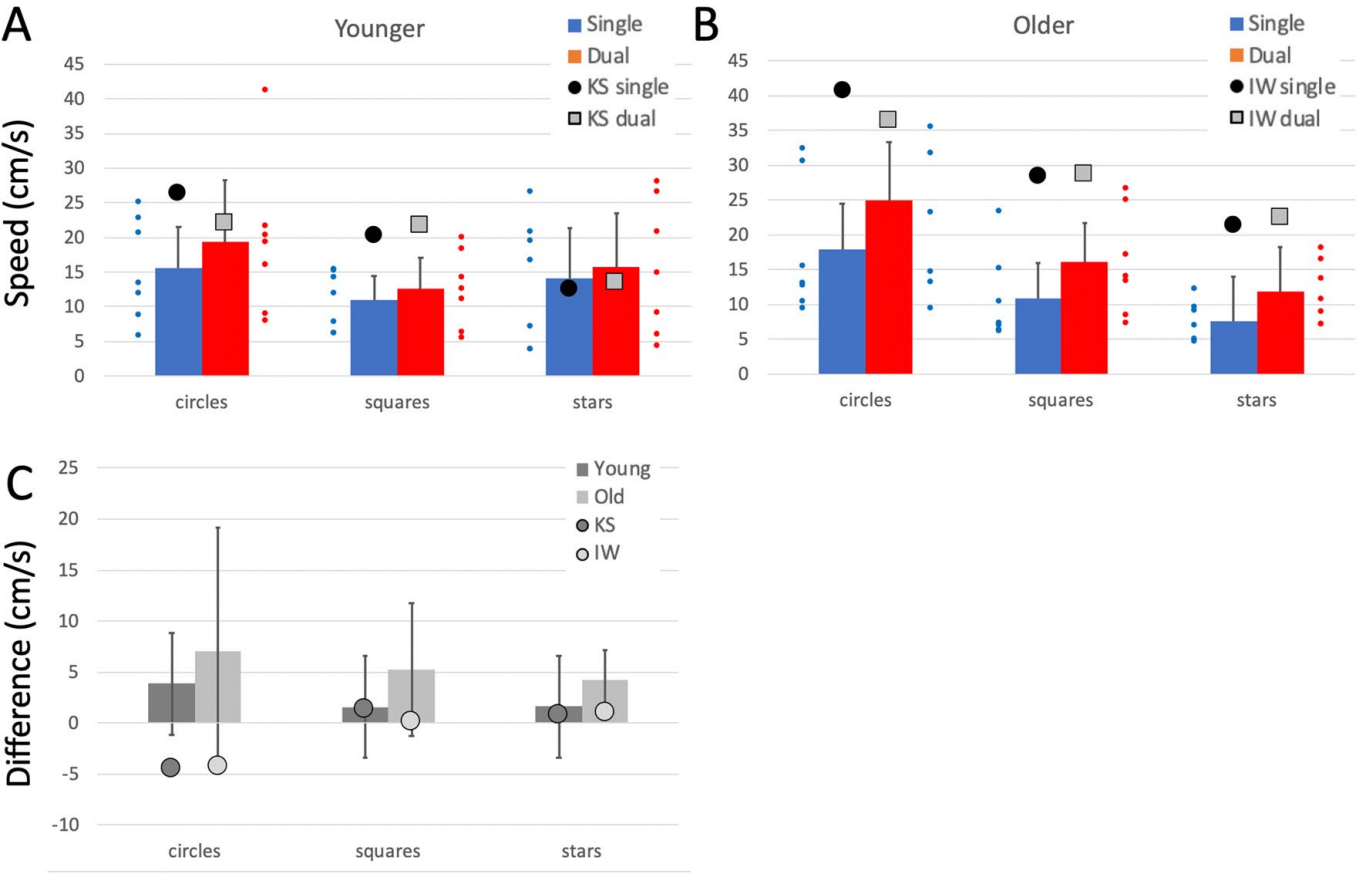
**a**, **b** Mean speed of drawing tasks (cm/s). **c** Difference in speed from single to dual-task conditions. Format is as in Fig. 4

Comparison of the control groups’ performance across the three shapes, with dominant and non-dominant hand, and with and without the dual task is presented in Supplementary Materials. The group data for their dominant hands, in single and dual tasks, are included in Figs. 4, 5 and 6 as bar plots.

#### Deafferented participants

##### Pathlength per cycle

Both deafferented participants drew larger shapes than controls (Fig. 4a, b; *q*´ > 3.03, *p* < 0.001 for all shapes), suggestive of the larger shapes drawn by the controls using their less practiced, non-dominant hand. Under dual-task conditions, KS’s circles and squares increased in size (Fig. 4a, c, see also Fig. 3), whereas, controls did not alter the size of their circle and square drawings; hence the pattern of changes across the three shapes for KS was different from that of the controls (*Q*´ = 11.33, *p* = 0.004; Fig. 4c). In contrast, under the dual-task conditions, IW drew smaller squares and larger stars (Fig. 4b), but always still larger than controls; the pattern of change for IW was not significantly different from his control group (*p* = 0.42; Fig. 4c).

##### Curvature

For a drawn (and therefore imperfect) circle, the curvature measure is dominated by the radius of the drawn shape, whereas, curvature differences in drawn squares and stars are more informative about the sharpness of the corners. So, while the shapes were somewhat larger, it is of note that both IW and KS had low curvature measures (rounded corners) compared to controls (Fig. 5), that were significant in the case–control comparisons for every condition (*q*´ < –3 .29, *p* < 0.001) except when KS drew stars/diamonds in the single task conditions: here the difference was a trend (*q*´ < – 1.45, *p* = 0.074). The reduction in curvature under dual-task conditions was smaller for IW and KS than for the controls (Fig. 5c), but the significance of these differences was marginal.

##### Path speed

IW was significantly faster than his control group in all conditions (*q*´ > 2.02, *p* < 0.022) while KS was faster for the simpler circle and square shapes (*q*´ > 2.84, *p* < 0.002). As was true for controls, both deafferented subjects showed a decrease in speed as shape complexity increased (Fig. 6).

Comparing the change in drawing speed under the dual task (Fig. 6c) highlighted a significant difference between KS and her controls (*Q*´(2) = 6.73, *p* = 0.034) because she slowed noticeably for the circle drawing (*q*´ = 2.63, *p* = 0.004). Speed dropped non-significantly in the other two conditions (*p* > 0.34). IW showed a similar pattern, slowing only for the circle drawing (*q*´ = 1.79, *p* = 0.037), but the factorial difference compared to the controls was not significant (*Q*´(2) = 3.39, *p* = 0.18), possibly reflecting high variance in the older control group.

##### Goodness of fit

Under single task conditions, IW was less consistent than controls in circle/jellybean drawing under single task conditions (*q*´ = − 3.29, *p* < 0.001), but more consistent for the squares/triangles and stars/diamonds (*q*´ > 2.37, *p* < 0.009); the older controls were poor at repeating the star shape. KS was also less consistent than her controls for circles and squares (*q*´ < − 2.02, *p* < 0.022) and tended toward greater consistency at the stars/diamonds (*q*´ = 1.34, *p* = 0.09).

Under dual-task conditions, KS showed a small increase in accuracy for circles and squares that was not seen in the controls (*q*´ < − 2.99, *p* < 0.001); she showed a small, insignificant decrease when drawing stars (*p* = 0.22); hence the factorial effect was significant (*Q*´(2) = 8.65, *p* = 0.013). IW also increased accuracy for all three shapes under the dual task, whereas, the older controls only improved for the squares drawing. This difference reached significance for circle drawing (*q*´ = − 2.27, *p* = 0.011); however, overall the factorial difference was not significant (*Q*´(2) = 1.75, *p* = 0.42).

##### Summary

Under dual-task conditions, controls showed increased path length and speed but reduced curvature (i.e. sharpness at the corners dropped) and reduced accuracy, effects that were exacerbated by use of the non-dominant hand (Supplementary Materials). These changes were most noticeable for the more complex shapes. Of note then, the two test participants, even under single task conditions, drew shapes larger, faster and with more rounded corners than controls; effects that are indicative of less precise motor control.

Under the dual task, IW and KS showed relatively small differences in speed and curvature for the complex shapes, but both slowed from high speed while drawing circles; KS also increased the size for circles and squares. This suggests that the addition of the cognitive load had differential effects: it negatively impacted drawing of the complex shapes for controls, but did not affect their drawing of circles, which were thus more automatic. But for KS and IW, drawing the complex shapes—which we presumed demanded high levels of control—continued with high accuracy scores despite the verbal echoing challenge, suggesting somewhat paradoxically that they maintained this control. The greatest change for both IW and KS was for the circles, which slowed during the dual task. This result may hint at a difference of automaticity for simple versus complex shapes but may also point to some factors not currently considered. It remains to be tested whether the shift in balance of their effort between the drawing task and the verbal echoing task was the same or opposite to that of the controls. Additional experiments are needed in which the dual task is either more demanding, or can be quantified, or both.

### Experiment 2—writing

#### Controls

**Fig. 7:**
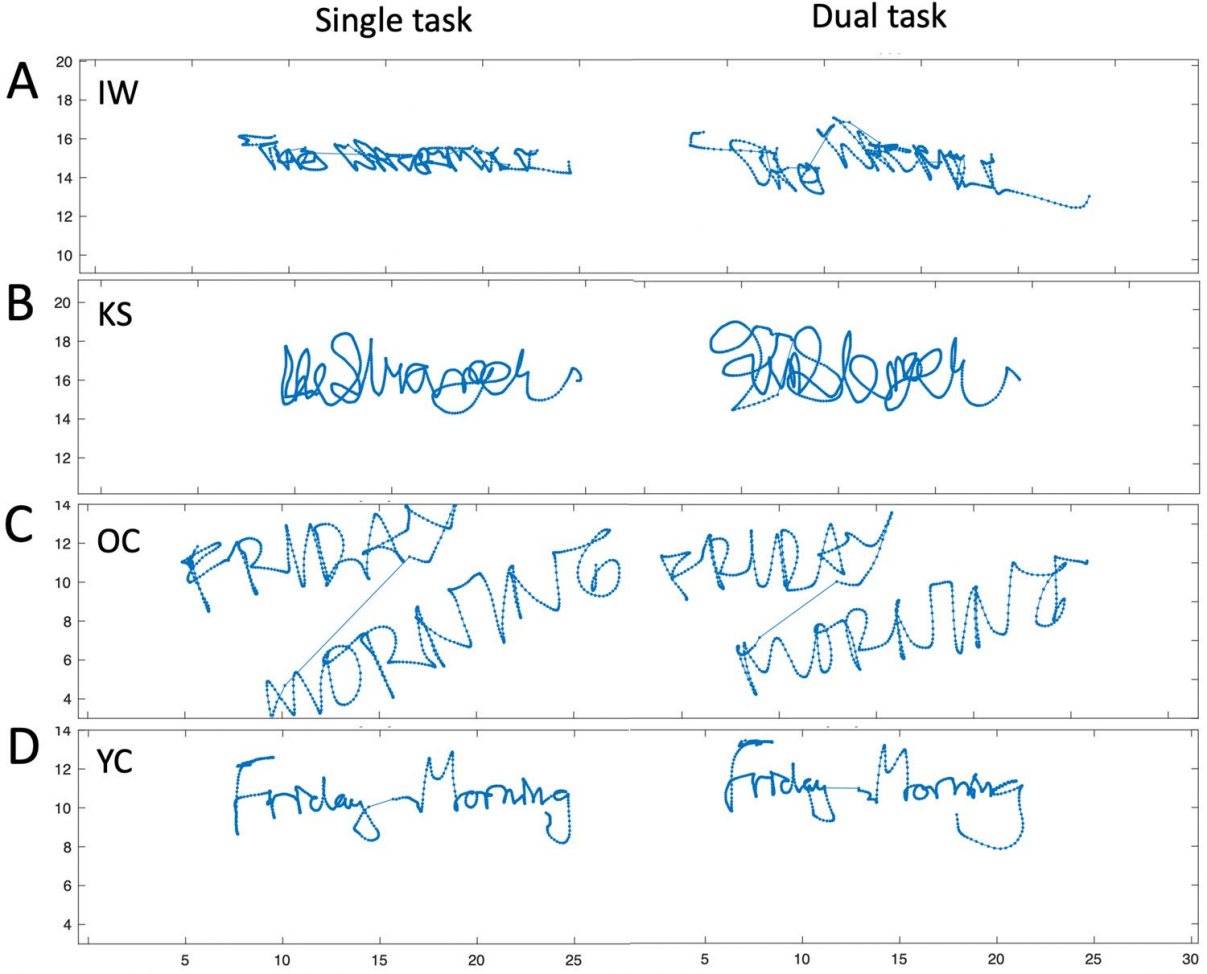
Examples of handwriting with and without the dual task. **a** IW writing ‘The Watermill, **b** KS writing ‘the Stranger’, **c** an older control and **d** a younger control, both writing “Friday Morning”. The right column shows examples as they wrote with a dual cognitive task. Note that the dual task caused more rounded lettering. For IW and KS, there was inaccurate letter placing, with much overlap, while for IW there was also a failure to keep on the horizontal. All axes are in cm

See Supplementary Materials for details; two examples of writing under single and dual-task conditions are shown in Fig. 7c, d.

#### Deafferented participants

Our aim was to see if there was evidence of automatic control for some common writing actions in IW and KS, as in the controls. Handwriting is not common for either IW or KS, but they do both write on occasion and do sign documents. IW writes in print, whereas, KS uses a cursive script. We hypothesised that if there was some automaticity, handwriting might be faster under the dual task, as in controls.

Comparison of the participants KS and IW with their respective control group was possible for the factors of word type (signature, name, phrase) and dual task effect; we did not challenge IW or KS with use of their non-dominant hands or with reverse direction writing. Notable changes in their writing under dual-task conditions included an increase in letter size and weaker placement on the page: note the vertical drift for IW, Fig. 7c, and the overlap of letters for both IW and KS, Fig. 7c, d.

##### Pathlength

**Fig. 8:**
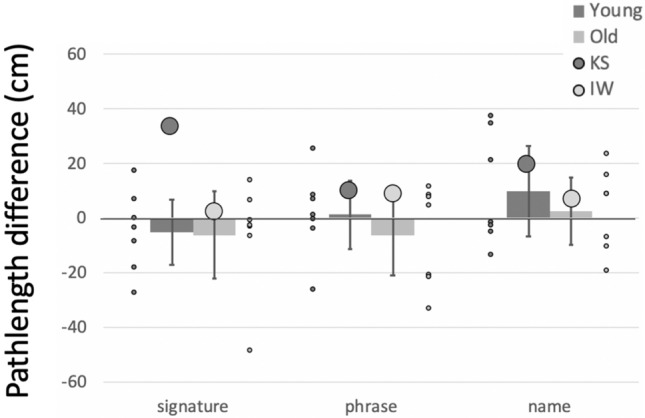
Mean pathlength difference for the writing tasks, measured in cm, between the single and dual-task conditions for writing tasks, for IW and the older control group. The younger control group mean difference is shown by the dark grey histogram (error bars are 95% confidence limits) and the individual data as small dark grey dots; the older controls are in light grey. Large dots are the mean data from KS (dark grey) and IW (light grey)

Pathlength is highly dependent on the size and length of the phrase being written. Hence, we are primarily interested in relative changes between writing conditions, in particular between the single and dual-task conditions (Fig. 8). In the factorial comparison of the single/dual task differences, we found KS differed from her control group across the main word factor (*Q*´(2) = 9.76, *p* = 0.008), which was driven by her production of longer traces under the dual task, especially for her signature (*q*´ = − 3.26, *p* = 0.001). Word pathlengths for IW were within, albeit on the high end of, the control range (*q*´ = 1.93, *p* = 0.27) and were not significantly changed by the dual task (*Q*´(2) = 1.03, *p* = 0.60).

##### Duration and path speed

**Fig. 9:**
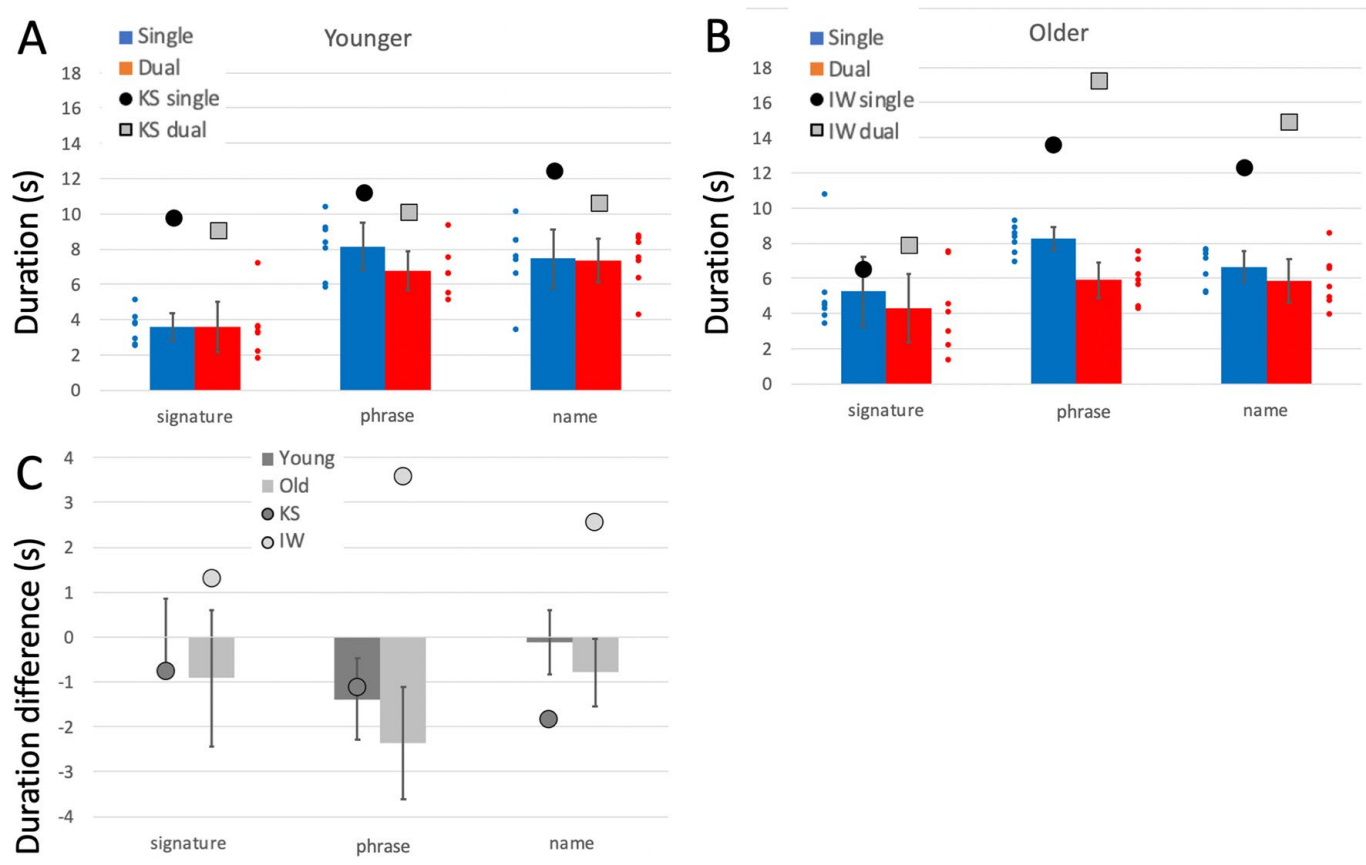
**a**, **b** Duration of writing tasks, in seconds. **c** Difference in duration from single to dual-task conditions. Format is as in Fig. 4

Metrics of duration and path speed were approximately reciprocal, and demonstrated that the two deafferented participants wrote more slowly than controls. Hence, the duration of each writing trial was significantly greater for both IW and KS than their controls (Fig. 9a, b; case–control comparisons *q*´ > 2.79, *p* < 0.003) except for IW’s signatures, and their mean speeds were significantly lower than their controls in most conditions (*q*´ < − 1.82, *p* < 0.034).

In the dual task, IW showed a significant increase in duration for each of the three writing tasks (9C; *q*´ < − 2.5, *p* < 0.006), and overall difference in pattern across the task factor (*Q*(1) = 11.93, *p* = 0.003). His reduction in speed with the dual task was opposite to his control group, who sped up (Supplementary Fig. 3; *q*´ > 1.84, *p* < 0.032) but the pattern difference across the tasks was not significant (*Q*´(2) = 0.13, *p* = 0.94).

For KS, the pattern of duration changes under dual-task conditions was significantly different from her controls across the three writing tasks (Fig. 9c; *Q*´(2) = 8.65, *p* = 0.013), with reduced duration for signature and name writing (*q*´ > 1.71, *p* < 0.043), but not for writing a phrase (p = 0.284). A similar pattern was seen for increase in path speed in the dual task (Supplementary Fig. 3; *Q*´(2) = 7.45, *p* = 0.024) which differed from the controls only for signature writing (*q*´ = − 2.37, p = 0.009; |*q*´|< 1.37, *p* > 0.085 for the other two).

##### Curvature

**Fig. 10:**
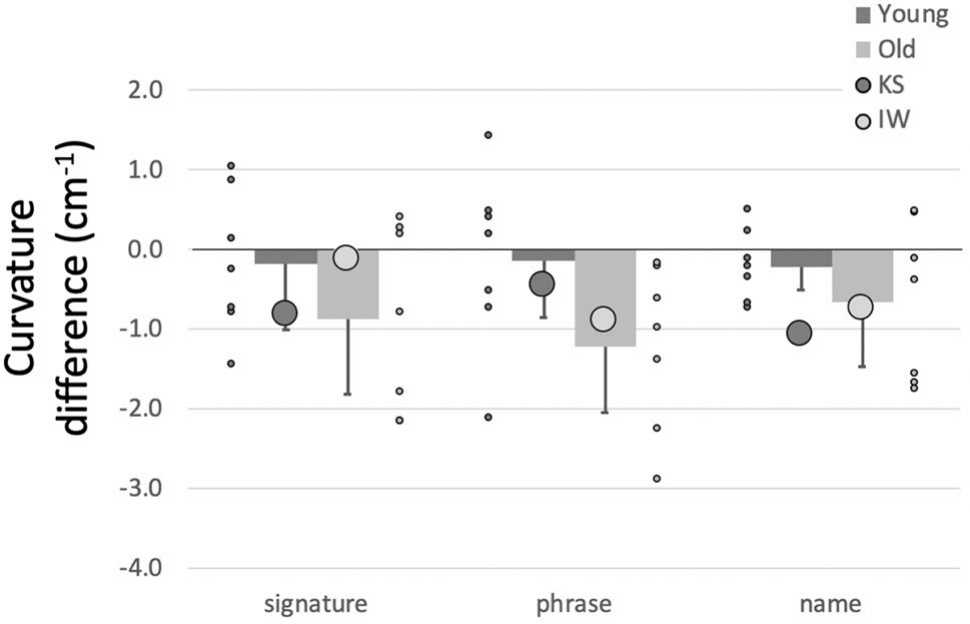
Difference in mean curvature of writing, from single to dual-task conditions. Format is as in Fig. 8

Mean curvature (the reciprocal of radius, cm^−1^) is also highly dependent on writing style, so only the relative differences across the three tasks with or without the dual task are important. While performing the dual task, all controls reduced curvature, with the older group doing so noticeably (Fig. 10). The reduction in curvature of IW’s signature under the dual task was less than for his control group (*q*´ = − 1.56, *p* = 0.059), but was equivalent to the controls when writing his name or a phrase (|*q*´|< 0.84, *p* > 0.2). For KS, who uses a cursive style, the reduction in curvature was greater under the dual condition for her signature and name writing (*q*´ > 1,71, *p* < 0.042) but not for writing words (*q*´ = 0.75, *p* = 0.22). In the factorial comparison, neither IW nor KS showed a significant difference across the words factor compared to the controls (*Q*´(2) < 1.69, *p* > 0.42).

##### Summary

The dual task reduced curvature and increased path speed, so reducing writing duration—and apparently greater facility of writing—for controls and for KS. Exceptions were the unchanged duration and path speed when the younger controls wrote their signature (see more in Discussion). KS and IW wrote much more slowly than did controls; unlike KS and either set of controls, IW slowed even further under dual-task conditions. In addition, we noticed that during the dual task, the amount of overwriting of individual letters (IW, KS) and vertical drift (IW only) increased; neither of these qualitative changes were seen in the controls. The dual task therefore had opposite effects on KS and IW, suggesting KS had access to some similar automaticity, as did controls, but which was unavailable to IW.

### Experiment 3—mirror-tracing

We previously showed that IW was significantly faster and more accurate than controls when mirror-tracing a smoothly curved shape, but was equally impaired in tracing shapes with sharp corners (Miall and Cole 2007). In the current experiments, we tested increasingly pointed shapes, from a smooth circle to a star. We hypothesised that KS and IW would be better than controls at the smoother shapes, and increasingly impaired, similar to controls, at the sharper-cornered shapes (Fig. 1).

#### Controls:

See Supplementary Materials, and see Supplementary Fig. 4 for examples of tracing of the shapes by members of the young and older control groups.

#### Deafferented participants

**Fig. 11:**
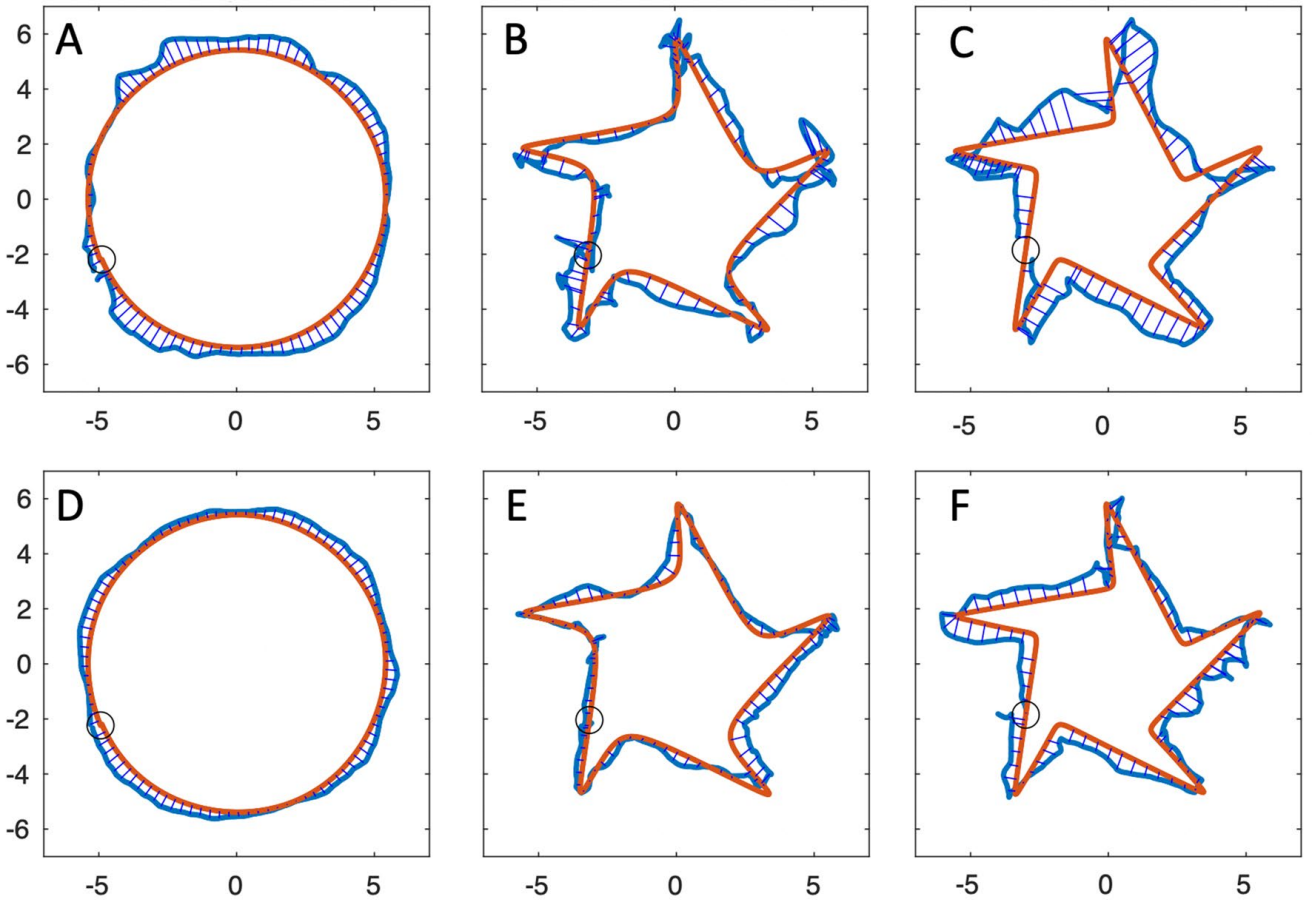
Mirror tracing by IW. **a**–**c** His first attempts at tracing shapes 1, 4, 5. **d**–**f** Attempts made after 10 min of training on other shapes. The small circle represents the start and end position. The fine blue lines linking the drawn shape (thick blue) and the template (red) are the errors to the nearest location, estimated at every 1/100 of the total path

**Fig. 12:**
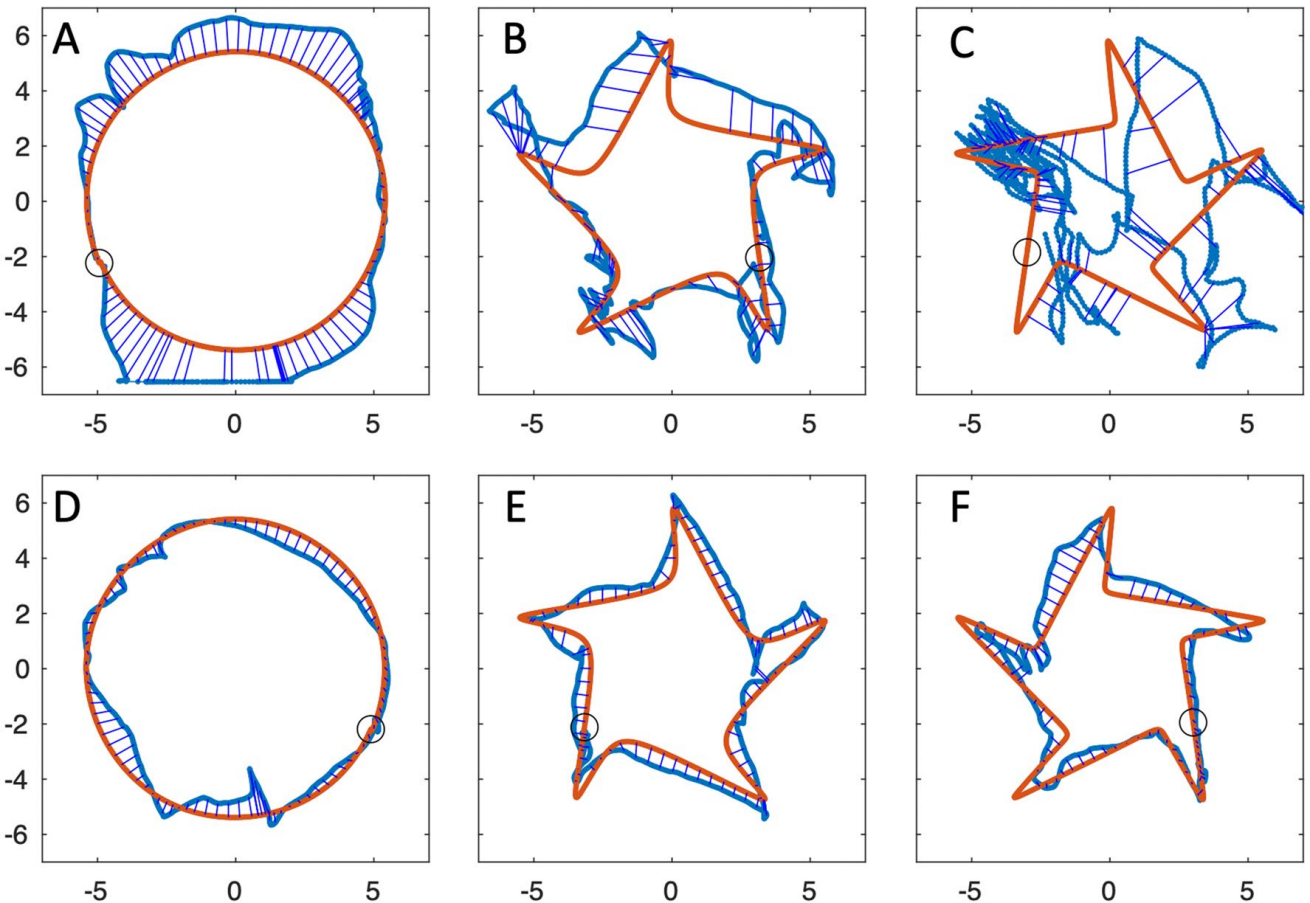
Mirror tracing by KS. **a**–**c** Her first attempts at shapes 1, 4, 5. Note the typical errors in direction, most obvious at the sharp corners in shape 5 (**c**), but also evident in the incorrect scaling of the circle (**a**). **d**–**f** attempts after 10 min of training on other shapes. Format as in Fig. 11

We were able to study the performance by KS and IW across the five shapes (Figs. 11a–c; 12a–c), but only with their dominant hands in the single task condition. We also report from trials where they traced directly on top of each template, without the mirror.

##### Duration

**Fig. 13:**
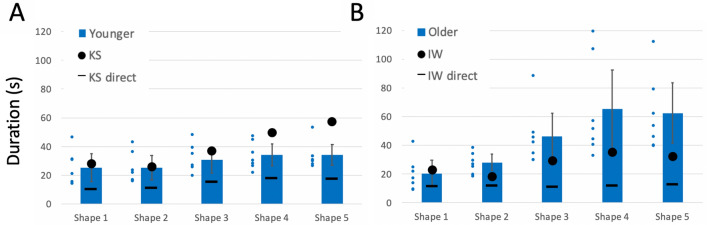
Duration for mirror drawing tasks in seconds. The blue bars are the control group means (*n* = 7); error bars are the 95% confidence limits. The black dots are the duration of mirror-tracing trials for IW (**a**) and KS (**b**) with the black horizontal bars representing their durations when directly tracing on top of the templates, without mirror reversal

KS was slower than the young controls in the three complex conditions (Shapes 3–5, Fig. 13a), significantly so for Shapes 4 and 5 (*q*´ > 2.98, *p* < 0.001). In contrast, IW was faster than the controls (*q*´ < – 1.92, *p* < 0.027) in all except the simplest shape (Fig. 13b; *q*´ = 0.60, *p* = 0.27). The factorial comparison was therefore significantly different for both KS and IW (*Q*´(4) > 14.89, *p* < 0.005), but reflected changes in opposite directions with respect to their control groups.

When directly tracing without the mirror, IW completed each shape in approximately constant time (10% increase from Shape 1 to 5; black horizontal bars, Fig. 13b), whereas, KS increased duration by 75% as the shapes became more complex (Fig. 13a).

##### Path speed

The control groups tended to slow down (from about 2.5 to 1.5 cm/s) as the complexity rose; both KS and IW maintained relatively constant path speed (both with average 2.0 cm/s). As a result, KS was on average faster than her control group, reaching 40% faster for Shape 5; IW was 30% slower than controls for the Shape 1 but about 20–40% faster for all others. Both KS and IW moved significantly faster when directly tracing without the mirror (2.9 cm/s for KS, 3.3 cm/s for IW).

##### Pathlength

The profiles for pathlength were similar to those of duration. IW did not differ from the controls (|*q*´|< 1.54, *p* > 0.061), whereas, KS’s pathlength was significantly longer in all five conditions (*q*´ < 3.29, *p* < 0.002), with a dramatic increase as the shapes became sharper. The factorial comparison was significant for her (*Q*´(4) = 58.18, *p* < 0.001) but not for IW (*Q*´(4) = 5.30, *p* = 0.26).

##### Error

Mean spatial error was approximately constant across all shapes. IW’s mean error tended to be similar to that of the controls (mean error for IW: 0.4 cm, mean for older group: 0.45 cm). In contrast, KS showed high errors (mean 0.74 cm), significantly greater than her control group across all 5 shapes (control mean 0.31 cm; *q*´ > 2.90, *p* < 0.002). The factorial comparison showed that KS’s difference from the controls varied across the 5 shapes, (*Q*´ > 10.77, *p* < 0.029) but there was little obvious pattern to these differences.

##### Summary

In mirror drawing, IW and KS differed from controls in opposing manners. They both moved the stylus faster than controls but only IW accomplished the tracing of the template in a shorter time than controls. KS took longer to complete the task, despite moving quickly, because of an increase in errors and consequently a large increase in pathlength; her greater duration was accentuated for the more complex shapes.

### Experiment 3A: effects of training

**Fig. 14:**
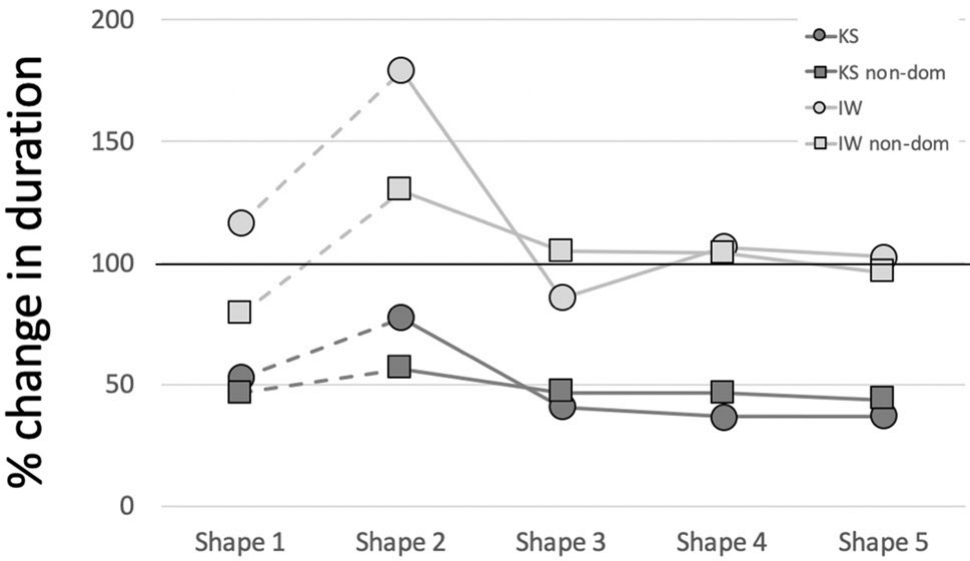
Change in tracing trial durations for IW (light grey) and KS (dark grey), after training. 100% reflects no change from the pre-training dominant hand conditions. Dots show the % change for the dominant hand while squares show the duration for the non-dominant hand relative to the pre-training dominant hand data. Note that Shape 1 was circular; the sharpness of corners increases from Shape 2 to 5

We examined the effect of a short training period on the mirror-tracing performance of IW and KS (Figs. 11d–f; 12d–f). In Fig. 14, the change in trial duration compared to their original performance (Fig. 13, black dots) is quantified. We show also how trials with their non-dominant hand, measured only after training, compared to pre- and post-training dominant hand data.

IW’s performance did not change dramatically after training with his dominant hand: trial duration was largely unchanged except for Shape 2 (Fig. 13a), which increased by ~ 75% mainly because he was relatively fast at this one condition before training; for Shapes 3–5, there was no effect of learning on duration. Path speed reduced somewhat (to 78% of the pre-training level, when averaged across Shapes 3, 4 and 5), as did pathlength (average 79%, and hence duration remained unchanged at 98%), and with this speed-accuracy trade-off, he achieved lower mean errors (60%).

In contrast to IW, KS showed a dramatic improvement, and after dominant-hand training she was significantly faster, reducing trial duration to about 50% of her pre-training performance (Fig. 14, dark grey). Averaged across Shapes 3,4 and 5, post-training speed increased to 132%, post-training duration was reduced to 38%, pathlength dropped to 51%, and mean error reduced to 42% of the pre-training levels (i.e. there was no trade-off between speed and accuracy, both improved). KS’s non-dominant hand duration, relative to the pre-trained dominant hand data, was indistinguishable from that of her post-training dominant hand. She achieved this while also reducing mean error, and pathlength by about 50% (data not shown).

While no directly comparable training data for the control groups exists, we saw no strong evidence for a practice effect, in the young control group, across the four testing sessions. The post-training performance for KS was notably better than the performance of the younger controls on their final test. For the older control group, there was a clear reduction in duration and increase in path speed, across the four sessions, especially for the more complex Shapes 3, 4 and 5. As mentioned above, such reductions were not evidenced by IW after training.

## General discussion

In these experiments we aimed to test whether KS, a woman who has lived her entire life without touch and proprioceptive input and sensation, may be more able to automate some of her actions than IW, a man who lost these as a young adult after a normal childhood. Because of their individual circumstances, neither of these two participants have had, or are likely to have, functional brain imaging studies. In the absence of that, information from behavioural tasks such as the writing and drawing tasks covered here assume more importance in determining the nature of any central reorganisation of their sensorimotor pathways.

Our hypothesis was that some simple actions might be automatic for KS, and so show similar changes to controls when faced with the cognitive challenge of a dual task (Posner and Snyder 1975; Logan 1979; MacMahon and Charness 2014). For KS, frequently performed actions such as wiping her mouth or driving her powered wheelchair are executed quickly and apparently without much conscious effort. In contrast, IW reports that all his movements require cognitive control, although he also states that after decades of practice, some actions are easier and require less oversight (Cole 1995). In general, these are actions such as gesturing, that do not involve interaction or manipulation in external space Cole (2016).

### Drawing shapes

In Experiment 1, we studied the repeated drawing of simple and more complex shapes. For controls, this was indeed sensitive to the dual task interference. In general, they tended to perform larger, faster and lower curvature (i.e. blunter) drawing when challenged with the dual task. Broeder et al. (2014) discuss that positive and negative dual task effects are common. A positive performance boost has been observed during expert sport tasks such as golf putting or soccer dribbling (Beilock et al. 2002, 2004) and in everyday automatic actions such as maintaining standing balance (Swan et al. 2004). In handwriting, disrupting attention enhances writing speed (Tucha et al. 2006) and the accuracy effects depend on writing expertise (MacMahon and Charness 2014); this may be because they reduce attention to the motor task—thereby lessening control. For controls, the drawing tasks used here likely represent an expert skill, particularly for the simpler shapes. When challenged by added cognitive load, controls showed kinematic changes consistent with looser control and so improved speed.

The deafferented subjects, IW and KS, were able to perform the drawing tasks well, at high speed and with reasonable accuracy under single task conditions; they were particularly fast compared to controls when drawing circles. Deafferented people have been previously shown to make simple repeated actions quickly, and with high temporal regularity (Gordon et al. 1995; Ingram et al. 2000; Messier et al. 2003). The observed increase in speed is predictable given the lack of corrective adjustments elicited by proprioceptive feedback. Without proprioceptive feedback, controls may also be more accurate when brief high force impulses are possible (Ingram et al. 2000). The same may be true of circle-drawing, where a relatively high force, high speed, oscillatory programme can be established and executed without much on-line control (Hollerbach 1981). It is of interest, then, that during the cognitive load, both IW and KS decreased their circle drawing speed, but did not change speed for squares or stars. This suggests that they can to some degree automate the production of these high speed and rapidly repeated circling shapes, but cannot maintain this under the dual task: unlike the controls, they are not “experts” at this task and show a negative dual task effect. It is important to note that even the simplest dextrous actions involving object manipulation, including holding and using a stylus, are difficult without somatic sensation (Rothwell et al. 1982; Cole 1995; Cuadra et al. 2019; Miall et al. 2019) and thus it may not be surprising that neither KS nor IW react to the dual task as experts.

### Writing tasks

Before discussing our findings, a word about differences in signature styles is warranted.

**Fig. 15:**
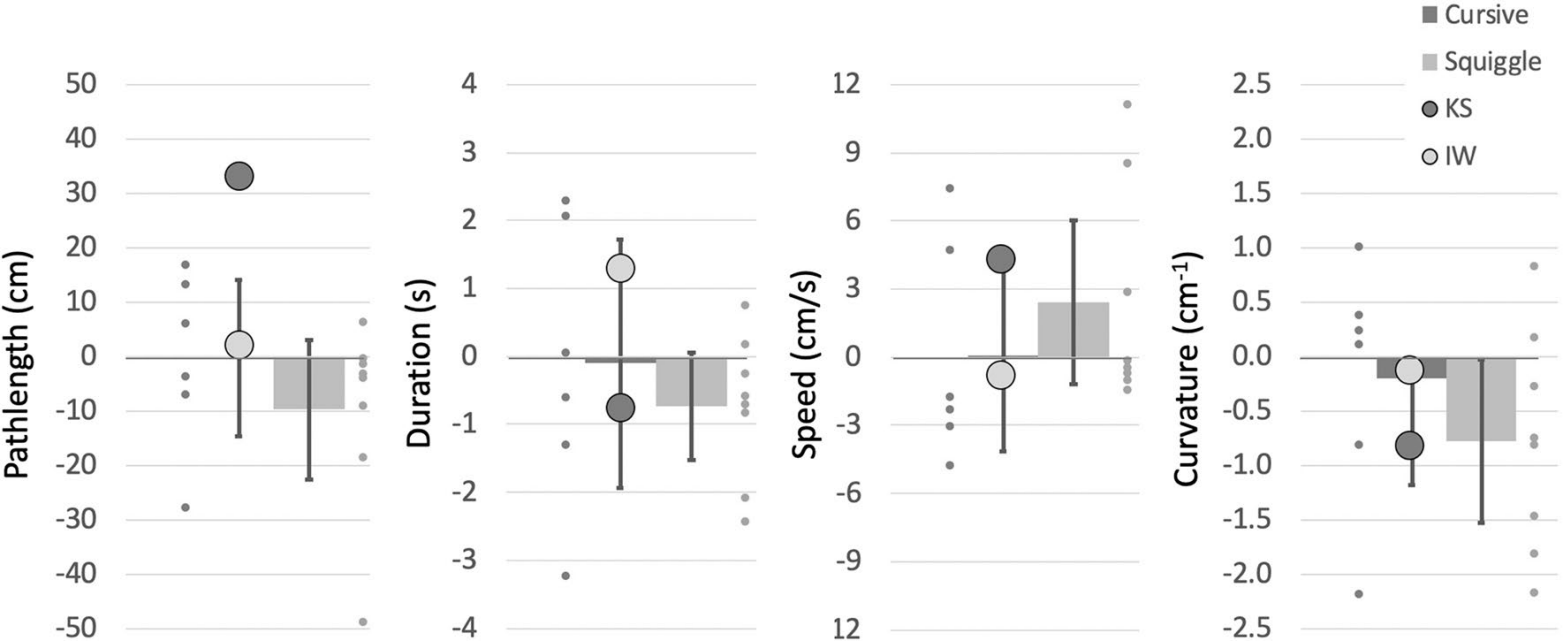
Changes in signature writing under dual-task conditions, with the controls classified by signature style (cursive, *n* = 6, vs squiggles, *n* = 8). Both IW and KS use a cursive style for signatures. Error bars are the 95% confidence intervals

The signatures of 8/14 controls were very quick “squiggles” whereas, the remaining six had more cursive, representative signatures; 4/7 of the older group and 2/7 from the younger group used a cursive style. By reclassifying the controls’ signatures as cursive vs. squiggles, regardless of age, we then saw changes under the dual task (Fig. 15) evident of automaticity for the “squigglers” who signed faster and with reduced curvature, whereas, the cursive group showed minimal changes. Hence, non-signature writing by the older group, and signing by the “squigglers,” were most altered under the dual task and, we suggest, are most automatic. The added cognitive demand of the dual task (or for the other writing tasks, the challenge of right-to-left or non-dominant hand-writing) was met by allowing looser motor control, shown by reduced curvature (less sharp corners and inflections), higher speed and shorter duration. These results are in line with those observed during free shape-drawing in the first experiment. As then, other research suggests that skilled and automatic action can tolerate (MacMahon and Charness 2014)—and even benefit from (Beilock et al. (2002, 2004), Broeder et al. 2014)—reduced focus on the motor task. One question we cannot directly address is the cost of reduced control on writing legibility: reduced curvature might reflect less legible scripts (Fig. 7) but other experiments would be needed to test this.

Both IW and KS wrote with lower speed and increased duration compared to their controls, suggesting that they needed to dedicate considerable attention and cognitive resources to writing. But under the dual-task conditions, only KS showed changes indicative of loosened control under the dual task constraint in the same way as did older controls, and significantly more than seen in the younger control group. These changes were greater when writing her signature, as was true for the “squigglers”. We take this as a sign that KS has automated her signature more than other writing tasks. For IW, the kinematic changes under the dual conditions tended to be opposite to the older controls across all three tasks, and were smallest for his signature. We interpret this as suggesting that he carefully controlled all writing, including his signature, and he did not allow (or could not adopt) looser more fluid, more automatic writing under the dual task.

Finally, we note that both IW and KS were able to execute individual letters well under dual-task conditions, but lost control of their placement on the drawing tablet (Fig. 7). This suggests separation of the control of shape from its placement, something that has been reported previously for IW in his hand gestures (Quaeghebeur et al. 2014) and is evident in normal participants when performing complex shape-copying tasks (Tchalenko 2009; Tchalenko and Miall 2017).

### Mirror tracing

Tracing shapes with mirror-reversed vision imposes the challenge of incongruence between visual input and proprioceptive input/motor planning (Lajoie et al. 1992; Miall and Cole 2007). Each abrupt change in target direction invokes a conflict between visual input and the motor plan as well as resulting proprioceptive input, so that deliberately corrected motor planning is needed to ensure movement is correct. Thus for both control participants and for IW, the dominant conflict appears to be between vision and motor planning (Miall and Cole 2007).

We speculate that tracing along smooth curves requires less planning. Thus, more continuously guided by visual feedback, controls experience an additional conflict between vision and proprioception, whereas, of course, the deafferented do not (Lajoie et al. 1992). Consistent with this, TMS-induced reduction in proprioceptive acuity in the fingers can enhance mirrored finger-tracking performance (Balslev et al. 2004). Hence, we predicted IW and KS would be faster and more accurate than controls tracking the smoother shapes (especially the circle, Shape 1) and smooth segments within the more complex shapes, while their performance would be more like that of controls for the sharpest corners (Shape 5).

The cognitive challenge of planning to trace around sharp corners was indeed confirmed to affect normal controls and both IW and KS, and was particularly obvious in the initial attempts by KS (Fig. 12c). Consistent with the suggestion that the difficulty is a cognitive planning conflict (Miall and Cole 2007), performance was poorer with the dominant hand relative to the non-dominant hand, suggesting conflict between automaticity and cognitive control, in so far as cognitive control must override the typical (non-mirrored) association between a visual representation of space and motor output (the visuo-motor mapping). Non-dominant hand performance is advantaged because the visuo-motor mapping is not as well-practiced (i.e. less automatic, less entrenched), meaning that less cognitive effort is needed to override it. We suggest therefore that the planning conflict is greatest when strong, well-entrenched predictive control is attempted, and is partially relieved under dual-task conditions (as tested for controls only), where attention must be shared, such that less cognitively-controlled visual feedback-guided tracking becomes more prominent.

IW completed the mirror-tracing trials more quickly and smoothly than did controls, suggesting he benefitted from the absence of visuo-proprioceptive conflict (Lajoie et al. 1992; Miall and Cole 2007), except for the simple circle (Shape 1) where a floor effect may have limited the differences. In contrast, KS showed a far poorer performance relative to her control group, worse in all measures, and performed particularly poorly on the more complex shapes (Shapes 3,4 and 5). Some of her individual trials showed severe “sticking” at the corners, as seen in controls (Supplementary Fig. 1). She appeared to reap no benefit from the absence of visuo-proprioceptive conflict.

The irregularity of KS’s circular mirror-tracing paths (seen also to a lesser degree for IW, Fig. 11a, d) are unlikely to be due to impaired motor control, i.e. regardless of mirror-reversal. When tracing under direct vision, without the mirror, both IW and KS showed dramatically increased speed and goodness of fit, along with reduced duration and pathlength. Their mean error was approximately halved relative to the mirror-tracing, albeit non-zero, reflecting small adjustments and corrections of their pen position. Hence with normal visual feedback, both were capable of accurate, fast and quite smooth tracing.

We suggest that the mirror task was more challenging for KS than IW, and indeed more challenging for her than controls, because her linkage between vision and action is particularly strong. In other words, because of her life-long condition, she may be even more dependent on tightly coupled unconscious visual input that serves motor functions as proprioceptive inputs do in controls. Hence, she found the mirror-reversal confounded all her initial attempts—and this affected even in the simplest circle shape (Fig. 12a, d). Neither IW nor GL (Lajoie et al. 1992) appear to have developed this unconscious link and so avoid the “visuo-proprioceptive” conflict (Lajoie et al. 1992; Miall and Cole 2007). Then, IW has a single conflict between vision and conscious motor planning, which is dependent on cognition, KS has an additional one between vision and movement control which is less accessible, or possibly entirely inaccessible, to her cognition. Controls also have two conflicts, between vision and motor planning, and between vision and proprioceptive feedback, and show rapid recalibration of the latter, feedback system with mirror-tracing practice (Lajoie et al. 1992). IW showed limited improvement with practise. It is true that IW has done a similar task before, but more than 15 years ago (Miall and Cole 2007) and with different shapes to trace. In contrast, KS’s rapid improvement with 10 min of training suggests she also recalibrated her visual feedback. In this KS shows a similar rapid adaptation to that seen in controls, though likely achieved by a very different mechanism (Lajoie et al. 1992).

Our data does not allow us to clearly separate within each trial segments of feedforward performance (when tracing corners) versus feedback-controlled tracing of straighter segments, but we speculate that it is these visuo-motor planning issues, rather than impaired feedback control (Gritsenko and Kalaska 2010; Kuang and Gail 2015), that led to KS’s initial errors on the smoothest shapes (e.g. Fig. 12a). As above, KS may be unusually dependent on (non-perceptual) visual feedback and experience a severe conflict between that and action, under mirror-reversed conditions, in a similar manner to normal participants’ proprioceptive-motor planning conflict.

The concept that visual information subconsciously guides movements is close to that termed ‘visual proprioception’ (Lee and Lishman 1975; Gibson 1979; Gallagher and Cole 1995). Visual proprioception refers to information that flows through visual pathways (but probably not through the ventral pathways that serve explicit visual perception) onto automatic somatomotor circuits. There is good evidence for fast and subconscious correction of posture and of reaching movements, based on such visual input (Lee and Lishman 1975; Pelisson et al. 1986; Franklin and Wolpert 2008; Franklin et al. 2012). This might be subcortical; one possibility is that the information travels from retina to superior colliculus (Reynolds and Day 2012), but it could also be cortical. The speed of the motor responses argues against a route via conscious declarative visual pathways. The “proprioception” portion of this term refers to an automatic, unconscious function. Much of (normal) somatosensory proprioception unconsciously guides movement.

It is unclear from existing studies whether the visual information is truly informative of the body position and speed etc. or whether it directly triggers visuo-motor updates to action. In other words, most experiments cannot separate evidence of visual coding of hand position from visual input of change in target location (in reaching actions) or a visual reference (in posture). Evidence of the former is provided by the contextual nature of the responses (with e.g. directionally tuned responses; Franklin and Wolpert 2008). Perhaps most compelling, however, is recent data from a companion paper (Miall et al. 2021), in which we show that KS has shorter verbal reaction times to visual stimuli that appear close to her hands. This, in normal participants, is an unconscious attentional bias to peri-personal space. IW does not have it, and KS shows it only when she can see her hands. We suggest this is an example of visual proprioception, independent of any visually-driven motor responses; it provides KS with information about hand position used to define her peri-personal space.

Underpinning KS’s purported visual proprioception might be developmental changes whereby visual information comprises a greater input to areas such as sensorimotor cortex and cerebellum. Adaptation to visual perturbations also involves sub-cortical (cerebellar) pathways (Martin et al. 1996; Robertson and Miall 1999; Block and Bastian 2012; Block and Celnik 2013; Yavari et al. 2016). Direct, visually mediated proprioception may thus account for KS’s better performance on the writing tasks (Experiments 1, 2), and support the increase in speed seen when she wrote under dual-task conditions. But it challenged her more under mirror-reversed conditions (Experiment 3). IW does not appear to have this subconscious ‘visual proprioception,’ and while he does adapt to visual changes, he may use cognitive strategies to do so (Taylor et al. 2010; Taylor and Ivry 2014).

In conclusion, our data suggest that her unique development may have endowed KS with visually originating, relatively automatic control of some simple actions, whereas, IW has replaced his missing somatosensation only through attention and cognitive control. Having said this, their persistence and creativity when faced with severe sensory deficits are extraordinary. Both continue to accomplish many daily tasks despite their profound impairments. They often accomplish tasks in ways viewed as unusual and laborious by controls; nonetheless, the tasks are accomplished.

## Supplementary Information

Below is the link to the electronic supplementary material.

Supplementary file1 (DOCX 2297 kb)

## Data Availability

The datasets generated or analysed during the current study are available from the corresponding author on reasonable request.
