# Supplementary material for: The role of somatosensation in automatic visuo-motor control: a comparison of congenital and acquired sensory loss
Source: Exp Brain Res. 2021 Apr 28;239(7):2043–61. doi: 10.1007/s00221-021-06110-y (PMC8282580; doi:10.1007/s00221-021-06110-y)
Supplement: Supplementary file 1 — Supplementary file1 (DOCX 2297 kb) [file 221_2021_6110_MOESM1_ESM.docx]

**Title**

The role of somatosensation in automatic visuomotor control: a comparison of congenital and acquired sensory loss

**Authors**

RC Miall, D Afanasyeva, JD Cole, P Mason

**SUPPLEMENTARY RESULTS**

**EXPERIMENT 1 – repeated drawing**

**Control participants:**

As expected, the shape complexity influenced all measures of performance, including increases in pathlength and speed, and reduction in curvature (Main text, Figures 4,5,6; F(2,24)> 16.75, p<.001). There was an interaction of shape and age-group for pathlength and speed (F(2,24)>4.3, p<.025) with the older group producing substantially longer pathlengths and lower speeds (Figures 4 and 6). The goodness of fit scores between successive cycles of drawing were close to one (>0.96) but declined for the most complex star shape to a mean across both age groups of 0.82, indicating greater variation from cycle to cycle (F(2,24)=20.18, p=.001, Greenhouse-Geisser corrected). There was an age difference (F(1,12)=5.40, p=.038) with the younger group being more consistent. Finally, the hand used also had a significant effect on curvature (F(1,12)=24.2, p<.001), with lower curvature with the non-dominant hand.

Turning to the effect of the dual task, we found that both pathlength and path speed increased (F(1,12)>5.98, p<.031; Figure 4, 6), while curvature decreased (F(1,12)=5.56, p=.036; Figure 5). There was also an interaction between dual task and shape for curvature (F(2,24)=3.80, p=.037), with reduced curvature for the more complex shapes under dual-task conditions.

**EXPERIMENT 2 – writing**

**Controls:**

We assumed that for the control groups, signature writing with the dominant hand would be automatic, whereas writing other phrases, writing with the non-dominant hand, or writing right-to-left would require differing amounts of cognitive oversight. The control participants performed each task with and without the dual echoing task. They wrote in their preferred style, most using a cursive script with their dominant hand (10/14 participants); four participants switched to a printing style with their non-dominant hand; four printed with both hands.

We found no overall effects of age group on the various metrics. Interactions of age with word type (signature, phrase and printed name) were noted for speed (Main text, Figure 9D) and pathlength (Supplementary Figure 1). However, we do not dwell on these differences as they do not pertain to the role of proprioceptive feedback but appear to reflect the larger/longer writing of phrases and names and concurrently, smaller and shorter signatures by the younger group compared to the older group (we return to this in the main text, Discussion).


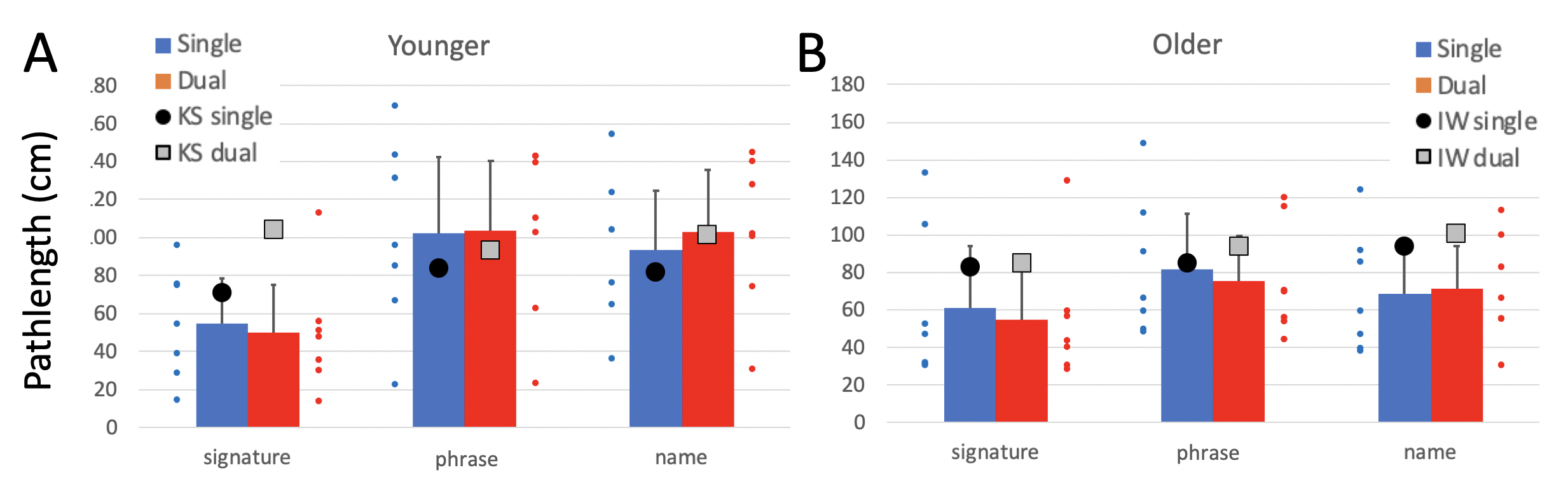


Supplementary Figure 1. A: Pathlength for writing tasks, measured in cm, for KS and the younger control group. B: IW and the older control group.

As expected, both control groups wrote faster, smoother and smaller with their dominant hands (duration, curvature, pathlength: F(2,12)>16.40, p<.002). They wrote more slowly (F(1,24)=17.04, p=.002) with greater curvature and total pathlength (F(1,24)>6.99, p<.023) when writing in the reverse direction.

Turning to the effect of the dual task, as a probe of the degree of automaticity of writing, we found a main effect on duration (Main text, Figure 9A-C), curvature (Supplementary Figure 2) and speed. Duration and curvature decreased (F(1,24)>15.08, p<.002) and speed increased (F(1,24)=22.69, p=.001). We also found interactions between the cognitive task and hand-use for curvature (F(1,11)=5.97, p=.033), and interactions between task, hand and direction for duration (F(1,11)=12.04, p=.005) and speed (F(1,11)=5.53, p=.038). In sum, the dual task increased writing speed, and reduced duration and curvature, with greater changes seen in the dominant hand. This is consistent with greater automaticity in more expert, dominant handwriting, expressed as faster smoother writing when attention is diverted to the dual task.


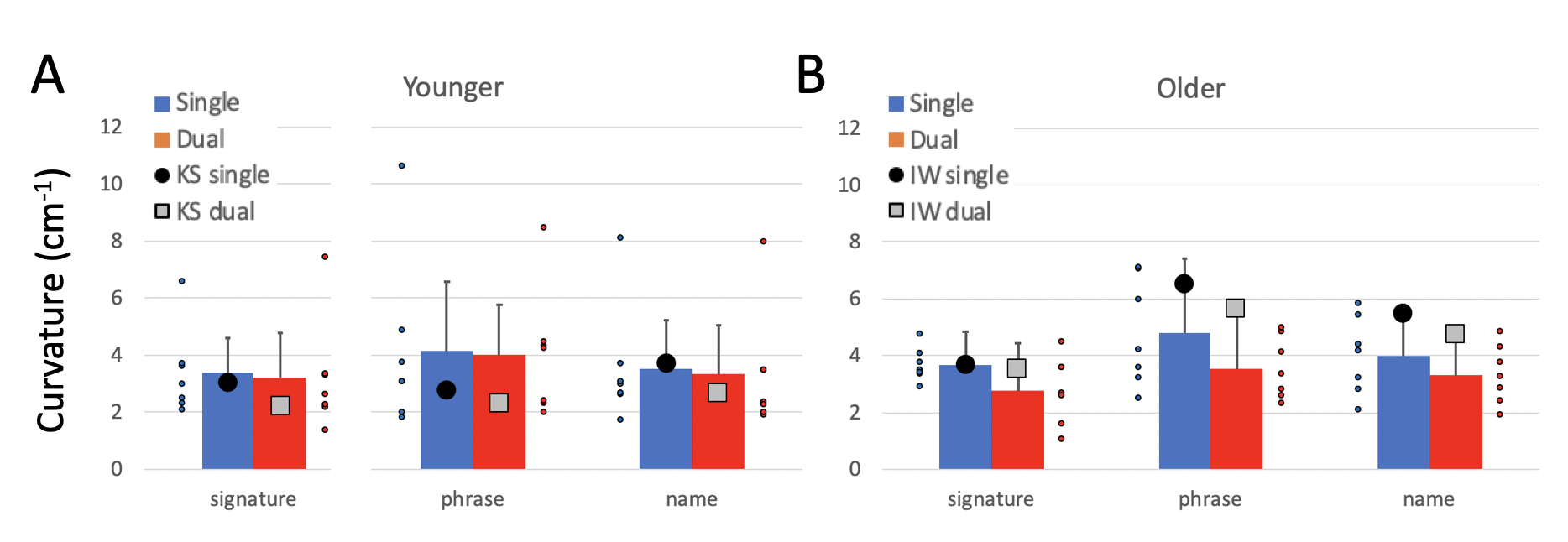


Supplementary Figure 2. A: Mean curvature, measured in cm^-1^, for KS and the younger control group. B: IW and the older control group.


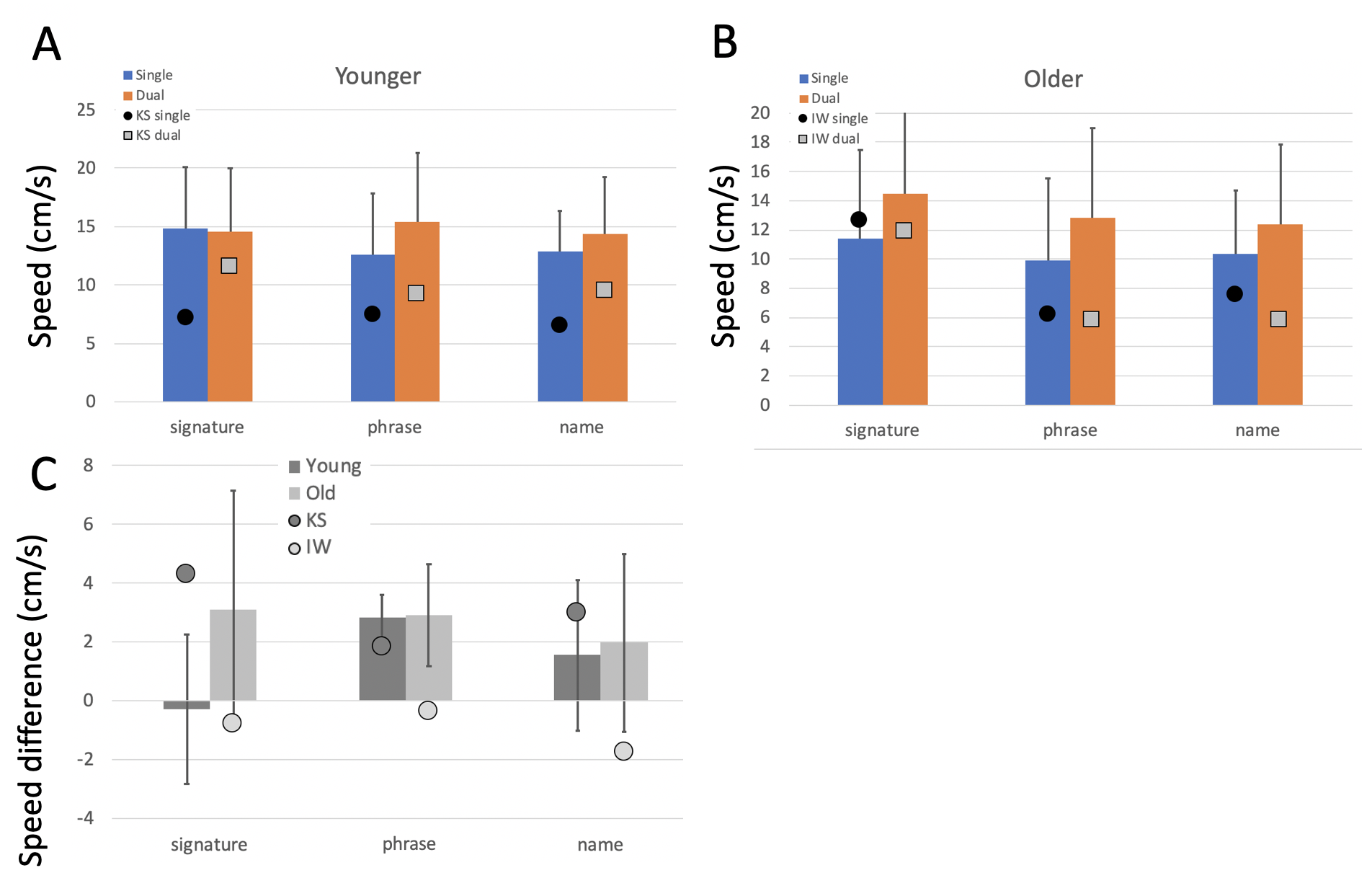


Supplementary Figure 3. A: Mean writing speed, measured in cm^-1^, for KS and the younger control group. B: IW and the older control group. C: difference in speed from single to dual task. Format is as in Main text, Figure 4.

There were interactions of age group with cognitive task for duration and curvature measures, with greater changes for the older group under the dual task, especially when writing their signature. In addition, interactions involving dual task and word type reached significance for path speed and duration (p<.022, Greenhouse-Geisser corrected), with a noticeable increase in path speed for the older group when writing their signature (Supplementary Figure 3C).

**EXPERIMENT 3 – mirror tracing**

**Controls:**

Supplementary Figure 4 shows examples from both age groups tracing Shapes 1 and 5, with the typical effects of mirror tracing – an inability to easily progress in the right direction with frequent laterally directed corrections (e.g. Supplementary Figure 4 B,E), and frequent sticking at the corners (Supplementary Figure 4 C,F). Tracing the diagonal segments also tended to be more challenging than those in vertical and near-horizontal sections.

As in Experiments 1 and 2, we first checked for any age-related differences in performance between the two groups. Duration and path speed were not significantly affected by age (F(1,12)<3.13, p>0.102). However, there were clear age-related differences in pathlength (F(1,12)=9.09, p=.011) and error (F(1,12)=9.80, p=.009) with the young group better in both instances.


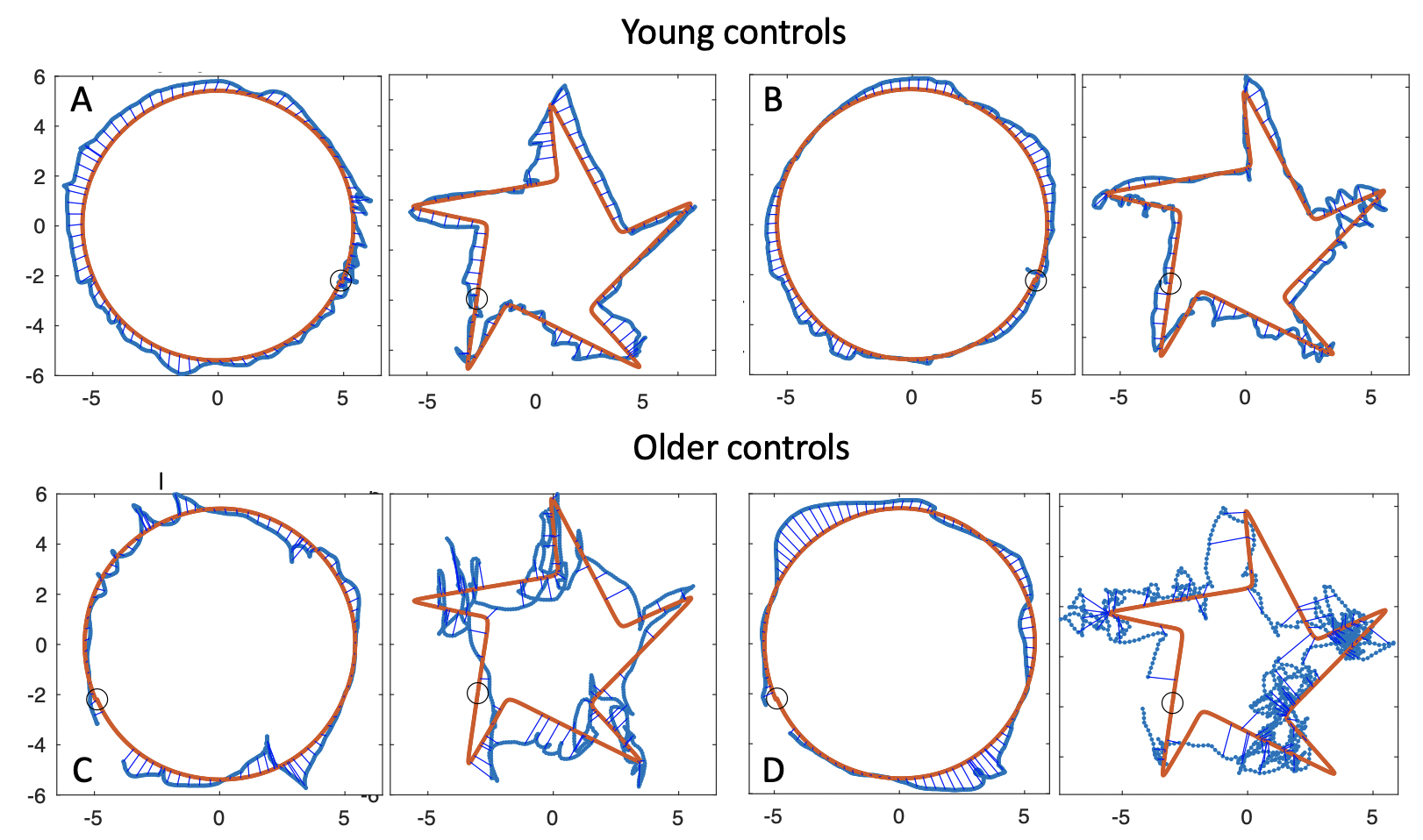


Supplementary Figure 4: Examples of mirror tracing by younger and older controls. To show the range of performance, we ranked the control participants by mean duration, and show their tracing of Shapes 1 and 5 by participants ranked 1, 6, 9, 14 (A-D).

As expected, we found very clear effects of the shape on duration, path speed and pathlength (F(4,48)>15.6, p<.0001). Duration increased (Main text, Figure 13), as predicted by our earlier study (Miall and Cole 2007), and was particularly affected by the shape factor in the older group. Pathlength rose, with a profile similar to duration. Path speed (not shown) decreased, dropping from an average 0.82 cm.s^-1^ to 0.46 cm.s^-1^. Error was lower for the younger group (F(1,12)=9.8, p=.009) and decreased systematically as complexity increased, from 0.4 cm to 0.33 cm (F(2.5,30.5)=4.57, p=.003, Greenhouse-Geisser corrected), presumably reflecting a speed-accuracy trade-off.

We next tested the effect of using the dominant and non-dominant hands. Duration and path speed were affected (mean duration 37.1s dropping to 24.8s for the non-dominant hand; mean speed rising from 1.7 cm.s^-1^ to 2.1 cm.s^-1^; (F(1,12)>14.97, p<.002); error was not significantly influenced (mean 0.38 cm dropping to 0.34 cm; F(1,12)<2.00, p>.18). There was also an interaction between hand and age group for duration (F(1,12)=11.27, p=.006) with the older group showing greater change as the shapes became complex than did the younger group. Finally, we found limited effects of the dual cognitive task, restricted to three-way interactions between the dual task shape and age-group for duration (F(4,48)=4.26, p=.028, Greenhouse-Geisser corrected). The older group improved most when tracing the complex shapes under the dual task conditions but this may be a practice effect, as the dual task (and non-dominant hand) followed test with the dominant hand). The older group was still slower than the younger group, who showed much smaller differences across the 4 trials.

To summarise, both groups of controls showed an advantage tracing smoother shapes (Shapes 1 and 2) and most difficulty with the sharper corners (Shapes 3, 4 and 5). But even on smooth diagonals they showed the typical effects of mirror tracing, being unable to easily progress in the right direction with frequent laterally directed corrections. These errors and hesitations increased trial duration, pathlength and mean error, as well as reducing mean path speed. Perhaps counterintuitively, these effects were greater for the dominant hand, suggesting the planning conflict is greater for the more frequently used writing hand, which may have stronger central control, and were relieved under dual task conditions, where attention must be shared.
